# Epigenetic Regulation of DAPK1 and Netrin‐1 Drives Diabetic Encephalopathy

**DOI:** 10.1002/advs.202502535

**Published:** 2025-08-11

**Authors:** Yang Zhou, Jia‐Xin Kou, Kai Zheng, Zi‐Xuan Guo, Hong‐Wei Fan, Wen‐Lian Li, Lu‐Lu Chu, Jing‐Wen Yin, Li‐Jie Liu, Nadezhda Brazhe, Zhi‐Gao Xiang, Feng Hu, Kai Shu, Ling‐Qiang Zhu, Dan Liu

**Affiliations:** ^1^ Department of Pathophysiology School of Basic Medicine Tongji Medical College Huazhong University of Science and Technology Wuhan Hubei 430030 China; ^2^ Department of Geriatrics Tongji Hospital Tongji Medical College Huazhong University of Science and Technology Wuhan Hubei 430030 China; ^3^ Department of Anesthesiology, Tongji Hospital, Tongji Medical College Huazhong University of Science and Technology Wuhan Hubei 430030 China; ^4^ Faculty of Biology M.V. Lomonosov Moscow State University Moscow 119234 Russia; ^5^ Department of Neurosurgery Tongji Hospital Tongji Medical College Huazhong University of Science and Technology Wuhan Hubei 430030 China; ^6^ Department of Medical Genetics, School of Basic Medicine, Tongji Medical College Huazhong University of Science and Technology Wuhan Hubei 430030 China

**Keywords:** DAPK1, diabetic encephalopathy, Netrin‐1

## Abstract

Diabetic encephalopathy (DE) is a severe complication of diabetes characterized by cognitive impairment and synaptic dysfunction, while the underlying mechanisms are not clear. Here, a critical role is identified for death‐associated protein kinase 1 (DAPK1) in DE pathogenesis using transgenic and streptozotocin‐induced diabetic mouse models. Elevated DAPK1 expression in hippocampal excitatory neurons correlates with cognitive deficits, increases neuronal apoptosis, and disrupts synaptic plasticity. Conditional knockout of DAPK1 in CaMKII‐positive neurons significantly mitigates these pathological features, improving cognitive performance and synaptic function. Mechanistically, it is demonstrated that reduced hippocampal microRNA (miR)‐216a‐5p levels in diabetic mice lead to DAPK1 upregulation. Furthermore, DAPK1 suppresses the expression of the neurotrophic factor Netrin‐1 (Ntn1) by phosphorylating hepatocyte nuclear factor 1 homeobox A (HNF1A), a key transcription factor. Silencing Ntn1 in wild‐type mice induces DE‐like symptoms, while intranasal administration of recombinant Ntn1 restores cognitive function and synaptic integrity in diabetic mice. These findings establish an miR‐216a‐5p/DAPK1/Ntn1 signaling axis as a critical driver of diabetes‐induced cognitive dysfunction and suggest Ntn1 as a promising therapeutic target for DE. Here novel insights into the molecular mechanisms are provided underlying DE, and the therapeutic potential of targeting DAPK1 and Ntn1 is highlighted to alleviate diabetes‐associated central nervous system complications.

## Graphical abstract

This study identifies a microRNA (miR)‐216a‐5p/death‐associated protein kinase 1 (DAPK1)/hepatocyte nuclear factor 1 homeobox A (HNF1A)/Netrin‐1 signaling axis in diabetic encephalopathy (DE). In leptin receptor‐deficient (db/db) and streptozotocin (STZ)‐induced diabetic mice, reduced miR‐216a‐5p increases DAPK1 expression, disrupting HNF1A binding to the Netrin‐1 promoter and suppressing its transcription. This cascade results in synaptic degeneration, neuronal apoptosis, glial activation, and cognitive impairment.

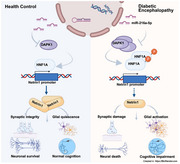

## 1 Introduction

Diabetes mellitus (DM) is the most common chronic and serious metabolic disease, characterizing by a hyperglycemia caused by absolute or relative insulin deficiency.^[^ ^1^, ^2^, ^3^, ^4^ ^]^ According to the International Diabetes Federation (IDF), the number of diabetes patients is ≈537 million around the world. By 2030, the number of diabetes patients will increase to 643 million, and it will increase to 783 million by 2045.^[^ ^5^ ^]^ Diabetes is related to a large number of long‐term complications influencing the kidneys, eyes, heart, and a variety of structural and functional deficits within the peripheral and central nervous systems (CNS).^[^ ^3^, ^6^, ^7^, ^8^ ^]^ Moreover, there are brain's structural and electrophysiological abnormalities giving ample justification to accept that cognitive functions may be disabled in DM patients.^[^ ^3^ ^]^ In the 1980–1990s, adults with DM were found to have moderate impairment of learning and memory,^[^ ^9^, ^10^ ^]^ thought to be associated with chronic hyperglycemia.^[^ ^11^ ^]^ A number of recent studies indicate that DM patients have a significantly higher risk of learning and memory disorders than nondiabetic people.^[^ ^12^, ^13^ ^]^ In addition, diabetes has been reported to cause cognitive impairment, and can even increase the risk of Alzheimer's disease.^[^ ^14^, ^15^, ^16^, ^17^ ^]^

Diabetic encephalopathy (DE) develops through complex interactions between chronic hyperglycemia, insulin resistance, and neuroinflammation.^[^ ^18^ ^]^ Persistent hyperglycemia triggers oxidative stress via reactive oxygen species (ROS) overproduction and activates the polyol pathway, leading to neuronal dysfunction.^[^ ^19^ ^]^ Insulin signaling impairment, evidenced by reduced p‐Akt and glycogen synthase kinase‐3 beta (GSK‐3β) activity, exacerbates apoptosis and synaptic damage.^[^ ^20^ ^]^ Neuroinflammation plays a central role, with elevated tumor necrosis factor‐alpha (TNF‐α), Interleukin‐6 (IL‐6), and receptor for advanced glycation end‐products/ nuclear factor‐kappa B (RAGE/NF‐κB) activation promoting Aβ accumulation and brain‐derived neurotrophic factor (BDNF) reduction.^[^ ^21^, ^22^ ^]^ These changes cause structural brain alterations, particularly in the hippocampus and frontal cortex, leading to cognitive decline.^[^ ^23^ ^]^ DE management now targets both metabolic and neurological aspects through multiple approaches. Antioxidants effectively reduce oxidative neuronal damage,^[^ ^24^, ^25^, ^26^ ^]^ while diabetes medications protect neurons via improved insulin signaling and anti‐inflammatory effects. Despite the progress achieved with antioxidant/anti‐inflammatory drugs in treating DE, the optimal dosage, treatment duration, and safety profiles remain to be further elucidated to ensure their therapeutic efficacy and clinical applicability as DE treatments. Regenerative options like stem cell and islet transplantation show tissue repair potential,^[^ ^27^, ^28^, ^29^, ^30^ ^]^ and nanomedicine (using polymeric/lipid nanoparticles) enhances CNS drug delivery,^[^ ^31^ ^]^ there are still pertinent problems that need to be solved.^[^ ^32^ ^]^ Unfortunately, as a chronic and serious metabolic disease, there are no effective measures to alleviate or delay cognitive dysfunction caused by diabetes currently. Therefore, exploring appropriate therapeutic targets and developing effective therapeutic drugs are of great clinical value to improve cognitive dysfunction caused by diabetes.

An increasing number of studies have shown that hyperglycemia can promote hippocampal cell death, such as apoptosis and autophagy, in the progression of cognitive dysfunction caused by diabetes.^[^ ^15^, ^16^, ^17^, ^33^ ^]^ Death‐associated protein kinase 1 (DAPK1) as a calcium/calmodulin (Ca^2+^/CaM)‐dependent protein kinase^[^ ^34^ ^]^ plays a key role in multiple modes of cell death.^[^ ^35^, ^36^ ^]^ Zhang et al. found that DAPK1 mediates synaptic dysfunction, leading to cognitive decline.^[^ ^37^ ^]^ We previously identified a direct binding of DAPK1‐kinase domain (DAPK1‐KD) to the DNA‐binding motif of the tumor suppressor protein‐p53, specifically blocking this binding in brain improved neurological functions and was effective against brain damages.^[^ ^38^, ^39^ ^]^ Structural and functional analyses identified that DAPK1‐KD orchestrated synaptic apoptosis, coupling developmental pruning to neurodegeneration‐associated synaptic loss.^[^ ^40^ ^]^ Previously studies from our laboratory have demonstrated that DAPK1 is directly interfaced with glutamate receptor channels‐*N*‐methyl‐d‐aspartate (NMDA) receptor NR2B subunit at extrasynaptic site, constituting a specific cell death signaling molecule.^[^ ^41^ ^]^ DAPK1 directly phosphorylated Tau at Thr231, Ser 262, and Ser 396, exacerbating tauopathy and neurofibrillary tangle formation in AD.^[^ ^40^, ^42^ ^]^ DAPK1 inhibition reversed Aβ‐induced synaptic plasticity deficits, demonstrating therapeutic potential for neurodegenerative diseases.^[^ ^42^ ^]^

The progression of diabetes was closely linked to systemic inflammation. Key factors include oxidative stress and the upregulation of inflammatory cytokines (e.g., TNF‐α and IL‐6), which impaired insulin sensitivity and contributed to metabolic dysfunction.^[^ ^43^ ^]^ Notably, inflammatory changes may precede the onset of overt diabetes, suggesting a causal role.^[^ ^44^ ^]^ Netrin‐1 (Ntn1), a laminin‐related protein, functions as a bifunctional neuronal guidance cue, attracting or repelling axons during migration.^[^ ^45^, ^46^ ^]^ While predominantly expressed in the CNS, it was also found in non‐neural tissues, including vascular endothelium, pancreas, liver, and kidney.^[^ ^47^ ^]^ Beyond its role in neurodevelopment, Netrin‐1 contributed to organogenesis (e.g., mammary glands and lungs), angiogenesis, and tumor progression.^[^ ^48^, ^49^ ^]^ Additionally, it regulated leukocyte trafficking, tissue repair, and inflammatory responses.^[^ ^47^, ^50^, ^51^, ^52^, ^53^ ^]^ Animal studies further demonstrated its antiangiogenic and cardioprotective effects, enhancing blood flow to ischemic tissue and mitigating ischemia‐reperfusion injury via nitric oxide signaling.^[^ ^54^, ^55^, ^56^ ^]^ Previous studies have revealed that elevated serum Netrin‐1 levels are significantly associated with impaired fasting glucose (IFG) and newly diagnosed type 2 diabetes, suggesting its potential role as a biomarker for early dysglycemia and insulin resistance.^[^ ^57^ ^]^ Tak et al. demonstrated that Netrin‐1 plays a kidney‐protective role in diabetic nephropathy, as evidenced by worsened renal injury in partially deficient mice and improved outcomes with recombinant Netrin‐1 treatment, mediated through Adora2b adenosine receptor‐dependent pathways.^[^ ^58^ ^]^ However, the potential role of DAPK1/Netrin‐1 in the DM‐induced cognitive dysfunction, remains elusive.

This study aimed to investigate the role of DAPK1 in diabetic cognitive impairment, with a focus on its neuronal specificity, upstream regulators, and downstream effectors. We hypothesized that DAPK1 hyperactivation in hippocampal excitatory neurons drives diabetic encephalopathy and that targeting the DAPK1/Netrin‐1 axis could mitigate these deficits. In this study, we report that cognitive dysfunction in transgenic and drug‐induced diabetic mice is positively correlated with elevated DAPK1 protein, accompanied by other pathologies such as neuroinflammation, apoptosis, and synaptic dysfunction (named diabetic encephalopathy). DAPK1 is mainly localized in Ca^2+^/calmodulin‐dependent protein kinase II (CaMKII)‐positive excitatory pyramidal neurons rather than inhibitory neurons of the hippocampus, where specific knockout of DAPK1 kinase domain in CaMKII^+^ neurons (DAPK1‐KD^−/−^ mice, abbreviated as KD^−/−^ mice) can significantly improve cognitive impairment and diabetic encephalopathy. We then identify microRNA (miR)‐216a‐5p acts as a post‐transcriptional regulator of DAPK1. As a potential target of DAPK1 regulation, the expression of growth factors and cytokines—Ntn1 in the hippocampus of db/db mice is significantly decreased. Silencing of Ntn1 induces the diabetic‐like cognitive dysfunctions and diabetic encephalopathy, while intranasal administration of recombinant Ntn1 slows down these symptoms in diabetic mice. Together, these gain‐ and loss‐of‐function studies reveal novel pathological events and mechanisms of the diabetic‐like cognitive dysfunctions and diabetic encephalopathy, which provides the basis for developing a novel therapeutic target for preventing the central nervous complications caused by diabetes.

## 2 Results

### 2.1 DAPK1 Levels Were Increased in the Brain of Diabetic Mice, with Diabetic Encephalopathy

We employed two classic diabetic mouse models in this study. The first model involved db/db mice, a type 2 diabetes model caused by a mutation in the leptin receptor gene, which is characterized by obesity, insulin resistance, and hyperglycemia.^[^ ^59^, ^60^ ^]^ The control group consisted of db/m heterozygous mice with the same genetic background, which do not exhibit diabetic phenotypes.^[^ ^59^ ^]^ The second model was induced by intraperitoneal injection of streptozotocin (STZ), a chemical that selectively destroys pancreatic β‐cells, thereby inducing type 1 diabetes.^[^ ^61^ ^]^ To ensure the accuracy of experimental controls, the control mice in the STZ group were injected with an equal volume of saline sodium citrate buffer (SCB) to account for nonspecific effects from the injection process.

First, we assessed diabetic symptoms in 6 month old db/db mice, as well as in C57 mice at 6 months of age, after STZ‐induced pancreatic damage administered at 2 months of age, and compared them with their respective control groups. We demonstrated that both the two diabetic mice models exhibited significantly elevated blood glucose levels (STZ group vs SCB group, 26.540 ± 1.331 vs 7.410 ± 0.439 mmol L^−1^; db/db group vs db/m group, 28.325 ± 0.566 vs 7.992 ± 0.566 mmol L^−1^) (Figure S1A, Supporting Information), as well as increased water (STZ group vs SCB group, 22.360 ± 0.745 vs 5.980 ± 0.300 mL day^−1^; db/db group vs db/m group, 21.858 ± 0.654 vs 6.100 ± 0.473 mL day^−1^) (Figure S1B, Supporting Information) and food intake (STZ group vs SCB group, 15.110 ± 0.504 vs 6.220 ± 0.237 g day^−1^; db/db group vs db/m group, 14.925 ± 0.518 vs 5.925 ± 0.289 g day^−1^) (Figure S1C, Supporting Information) compared to the control group. The body weight of db/db mice (52.394 ± 1.177 g) was higher than that of mice in the control group (23.429 ± 0.485 g); but this change was not observed in STZ‐induced diabetic mice (21.809 ± 0.705 g), which even showed a slight weight loss (Figure S1D, Supporting Information). To evaluate glucose tolerance and insulin sensitivity in diabetic mice, we performed oral glucose tolerance tests (OGTT) and insulin tolerance tests (ITT). We showed that diabetic mice had significantly elevated blood glucose levels after glucose loading and slower recovery, with a significantly higher area under the curve (AUC) compared to the control group (Figure S1E, Supporting Information), indicating impaired glucose tolerance and insulin resistance. The ITT results demonstrated that blood glucose levels decreased to less around 13.09% and 12.06% within 15–30 min of insulin injection in STZ mice and db/db mice separately. Both STZ and db/db mice failed to return to fasting levels within 1 h, displaying a profound whole body insulin resistance (Figure S1F, Supporting Information). Furthermore, diabetic mice exhibited reduced fasting insulin levels (STZ group, 3.135 ± 0.759, µmol mL^−1^; db/db group, 4.474 ± 0.818 µmol mL^−1^) (Figure S1G, Supporting Information), and their homeostasis model assessment of insulin resistance (HOMA‐IR) was significantly higher than that of the control group (Figure S1H, Supporting Information). These results indicate that the diabetic mouse models used in this study displayed diabetic‐like phenotypes.

**Figure 1:**
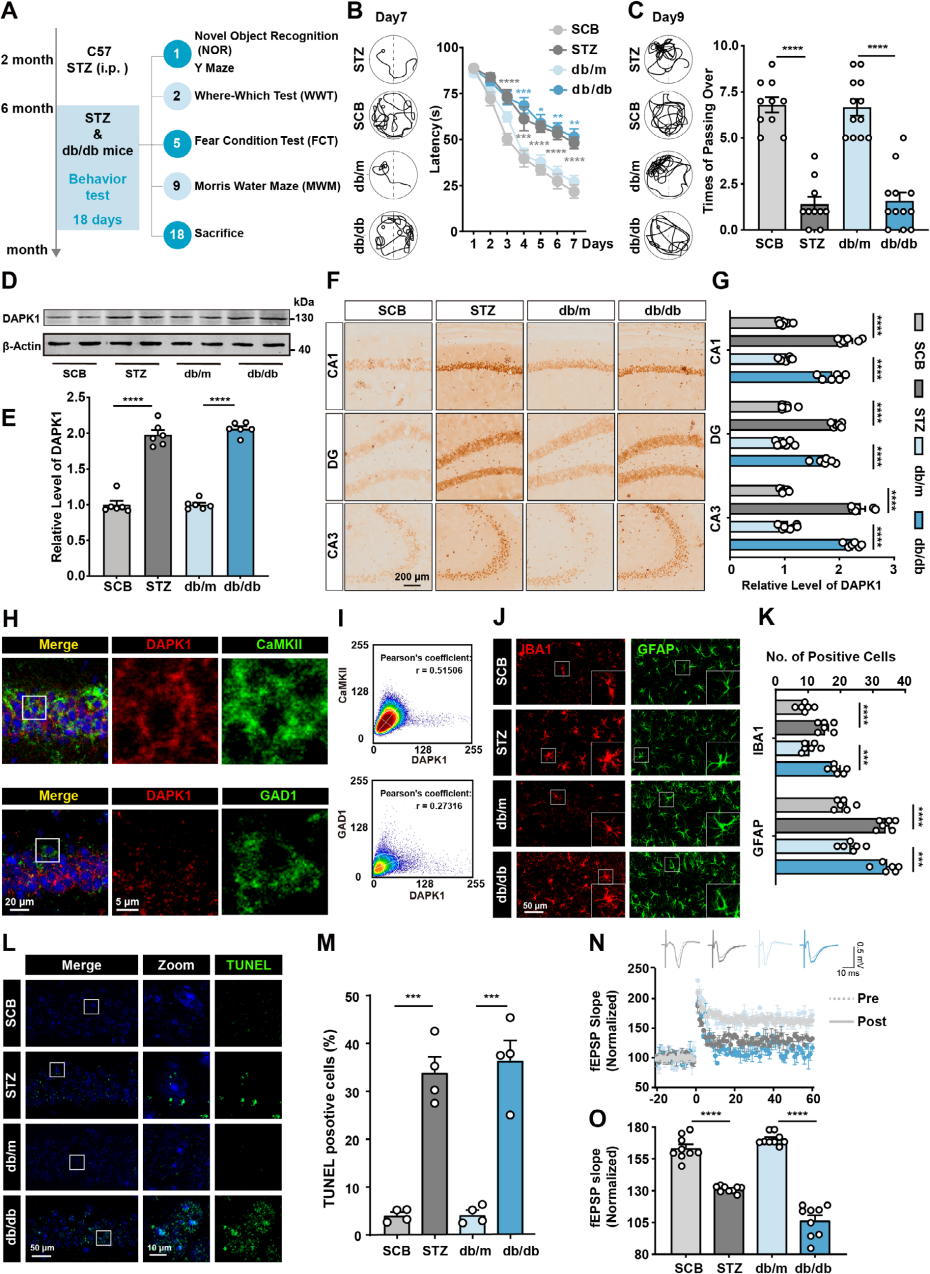
DAPK1 levels were increased in the brain of diabetic mice with diabetic encephalopathy. A) Schematic overview of diabetic mouse modeling and cognitive‐related behavior assessments. B,C) Representative swimming paths on day 7, B) latency to reach the platform from days 1–7, C) swimming paths on day 9 and number of crossing the platform region on day 9 in the Morris water maze task for diabetic groups (6 month old STZ‐treated mice and db/db mice) and control groups (6 month old SCB‐treated mice and db/m mice). *n* = 10–12 per group. D) Western blot analysis and E) quantification of DAPK1 protein expression relative to β‐actin in the hippocampus of diabetic and control groups. *n* = 6 per group. F) Representative images and G) quantitative analysis of immunohistochemical staining for DAPK1 in hippocampal subregions (CA1, DG, and CA3) of diabetic and control mice. *n* = 6 slices from 3 mice per group. H) Distribution of DAPK1 (red) in excitatory pyramidal neurons (CaMKII, green) and its absence in inhibitory neurons (GAD1, green). Nuclei were stained with DAPI (blue). Higher‐magnification images of the regions marked with white squares in the CA1 area are shown on the right. I) Colocalization analysis of DAPK1 with CaMKII (up) and GAD1 (down), showing overlay levels and Pearson's correlation coefficient. J) Representative images and K) qualification of microglia (IBA1, red) and astrocytic (GFAP, green) activation in the hippocampus of diabetic and control groups. *n* = 6 slices from three mice per group. L) Representative images and M) quantification of apoptotic cells in the hippocampus of diabetic and control groups, as indicated by TUNEL‐positive staining (green). Nuclei were stained with DAPI (blue). The regions marked with white squares in the CA1 area are shown at higher magnification on the right. The percentage of apoptotic cells was calculated with the number of TUNEL‐positive cells divided by the total number of DAPI‐labeled cells in the same microscopic field. *n* = 4 slices from three mice per group. N,O) Electrophysiological recordings of N) field excitatory postsynaptic potential (fEPSP) slope to evaluate the LTP at the CA3‐CA1 synapse. *n* = 9 cells from three mice per group. Data are presented as mean ± S.E.M., and statistical analysis was performed using an unpaired Student's *t*‐test or one‐way or two‐way analysis of variance (ANOVA), unless otherwise specified. ** *p* < 0.01, *** *p* < 0.001, and **** *p* < 0.0001.

Then, we used a series of behavioral experiments to assess changes in the cognitive abilities of mice (**Figure** **1**A). We evaluated the learning memory of these mice at 6 months old by new object recognition (NOR), Y maze, where–which test (WWT), fear condition test (FCT), and Morris water maze (MWM) tasks. We found that diabetic mice displayed longer latency on day 7 (STZ group, 48.190 ± 3.103 s; db/db group, 51.317 ± 4.453 s) to less frequency to cross the platform region on day 9 (STZ group, 1.400 ± 0.400; db/db group, 1.583 ± 0.452) during the probe trial of MWM (Figure 1B,C). The percentage of spontaneous alternation was significantly lower in diabetic mice (STZ group, 13.750 ± 4.241%; db/db group, 13.775 ± 3.113%) compared to the control group (SCB group, 69.937 ± 3.418%; db/m group, 64.167 ± 4.269%) in the Y maze (Figure S1K, Supporting Information). We also evaluated context‐place memory using the WWT and NOR, and the results showed that the diabetic mice had significantly lower recognition index compared to the control group, indicating impaired spatial memory performance (Figure S1M,O, Supporting Information). Freezing behavior in the FCT was also reduced (STZ group, 32.710 ± 3.172%; db/db group, 32.108 ± 33.416%) (Figure S1Q, Supporting Information), indicating impaired fear memory consolidation associated with diabetic conditions. Based on the above behavioral results, we conclude that both diabetic mouse models exhibit significant impairments in cognitive function.

DAPK1 is a Ca^2+^/CaM‐dependent serine/threonine (Ser/Thr) protein kinase,^[^ ^34^, ^40^ ^]^ whose function is involved in signaling pathways related to cell death,^[^ ^62^ ^]^ as well as the regulation of apoptosis^[^ ^63^ ^]^ and neuronal cell death.^[^ ^64^ ^]^ Research has demonstrated that DAPK1 activation in the hippocampus contributes to the impairment of learning and memory during aging by promoting the degradation of Caytaxin, a protein strongly linked to intellectual disability.^[^ ^65^, ^66^ ^]^ To understand the potential role of DAPK1 in diabetic encephalopathy, we then assessed its expression across different diabetic mouse models by using western blot analysis. We noticed that DAPK1 protein levels in the hippocampus of diabetic mice were significantly increased compared with controls, approximately twice that of controls (Figure 1D,E). Immunohistochemical staining further confirmed that DAPK1 expression was elevated in the hippocampal subregions CA1, dentate gyrus (DG), and CA3 of diabetic mice relative to controls (Figure 1F,G). We subsequently performed a correlation analysis between hippocampal DAPK1 expression levels and behavioral outcomes in mice. Our results showed that DAPK1 expression levels were positively correlated with the latency to reach the target platform in the Morris water maze and negatively correlated with the number of crossings over the target area (Figure S1I,J, Supporting Information). These findings suggest that higher DAPK1 expression is associated with impaired cognitive performance in mice. Similar correlations were observed between DAPK1 expression and other behavioral outcomes (Figure S1L,N,P,R, Supporting Information). To further investigate the interrelationships among different behavioral paradigms, we performed correlation analyses across various cognitive tests. The results revealed consistent positive correlations among behavioral outcomes, suggesting a shared underlying construct of cognitive performance (Figure S1S–AB, Supporting Information). For example, longer escape latency in the Morris water maze was significantly associated with lower freezing percentages in the fear conditioning test (*R* ^2^ = 0.4321, *p* < 0.0001) (Figure S1S, Supporting Information), reduced spontaneous alternation in the Y maze (*R* ^2^ = 0.7227, *p* < 0.0001) (Figure S1T, Supporting Information), and reduced discrimination indices in both the novel object recognition (*R* ^2^ = 0.4553, *p* < 0.0001) (Figure S1U, Supporting Information) and WWT (*R* ^2^ = 0.4969, *p* < 0.0001) (Figure S1V, Supporting Information). These findings indicate that diabetic conditions may impair multiple domains of cognition in a coordinated manner, and that behavioral deficits in one task can serve as a reliable predictor of broader cognitive dysfunction.

Using double immunofluorescence, we identified that DAPK1 was detected in neurons (Figure S2A, Supporting Information), and enriched in Ca^2+^/CaMKII‐positive excitatory pyramidal neurons but absent in inhibitory neurons (Figure 1H,I, Supporting Information). Western blot analysis revealed a significant reduction of CaMKII in STZ‐induced diabetic mice (1.000 ± 0.035) compared with nondiabetic controls (SCB, 0.906 ± 0.013), while GAD1 levels remained unchanged (SCB, 1.000 ± 0.019; STZ, 0.980 ± 0.029) (Figure S2B,C, Supporting Information). A correlation analysis showed a negative trend between DAPK1 and CaMKII expressions (*R* ^2^ = 0.3224, *p* = 0.0514), suggesting that DAPK1 may contribute to the downregulation of CaMKII in diabetes, although this relationship did not reach statistical significance (Figure S2D, Supporting Information). To test whether DAPK1 directly influences CaMKII expression, we analyzed STZ‐diabetic mice with conditional knockout of DAPK1. In STZ/KD^−/−^ mice, CaMKII levels were significantly higher than in STZ/KD^f/f^ littermates (STZ/KD^f/f^, 1.000 ± 0.018; STZ/KD^−/−^, 1.092 ± 0.021), indicating that DAPK1 deletion partially alleviates CaMKII downregulation (Figure S2E,F, Supporting Information). GAD1 expression was not affected by DAPK1 status, consistent with its unaltered levels in STZ mice (STZ/KD^f/f^, 1.000 ± 0.028; STZ/KD^−/−^, 1.012 ± 0.026).

We further assessed neuroinflammation, apoptosis, and synaptic plasticity in diabetic mice. Immunofluorescence staining revealed increased activation of microglia (ionized calcium binding adapter molecule 1, IBA1) and astrocytes reactivity (glial fibrillary acidic protein, GFAP) in the hippocampus of db/db and STZ‐treated mice compared to control, indicating heightened neuroinflammation (Figure 1J,K). Terminal deoxynucleotidyl transferase dUTP nick end labeling (TUNEL) staining, a commonly used method for detecting apoptotic cells, showed that STZ‐induced mice exhibited an increase in cell apoptosis, which was ≈29.81 ± 3.37% higher compared to the SCB control group. Similarly, db/db mice displayed an increase of about 32.21 ± 4.31% cell apoptosis compared to db/m group (Figure 1L,M). These findings indicate a significant increase in neuronal apoptosis in the hippocampus of diabetic mice. Then we recorded field excitatory postsynaptic potentials (fEPSPs) in the CA1 region by electrical stimulation of the mossy fiber tracks that reflect synaptic responses from neurons in the dentate gyrus. Compared with the control group, our results showed that the fEPSP slope of both STZ group mice and db/db group mice was notably decreased, which indicated that the synaptic plasticity was impaired in diabetic mice (Figure 1N,O). These results indicate that DAPK1 expression is increased in hippocampal excitatory neurons of diabetic mice, accompanied by hippocampal neuronal apoptosis and impaired synaptic plasticity.

### 2.2 Knockdown Hippocampus DAPK1 in Excitatory Neurons Rescued Diabetic Encephalopathy in Diabetes

**Figure 2:**
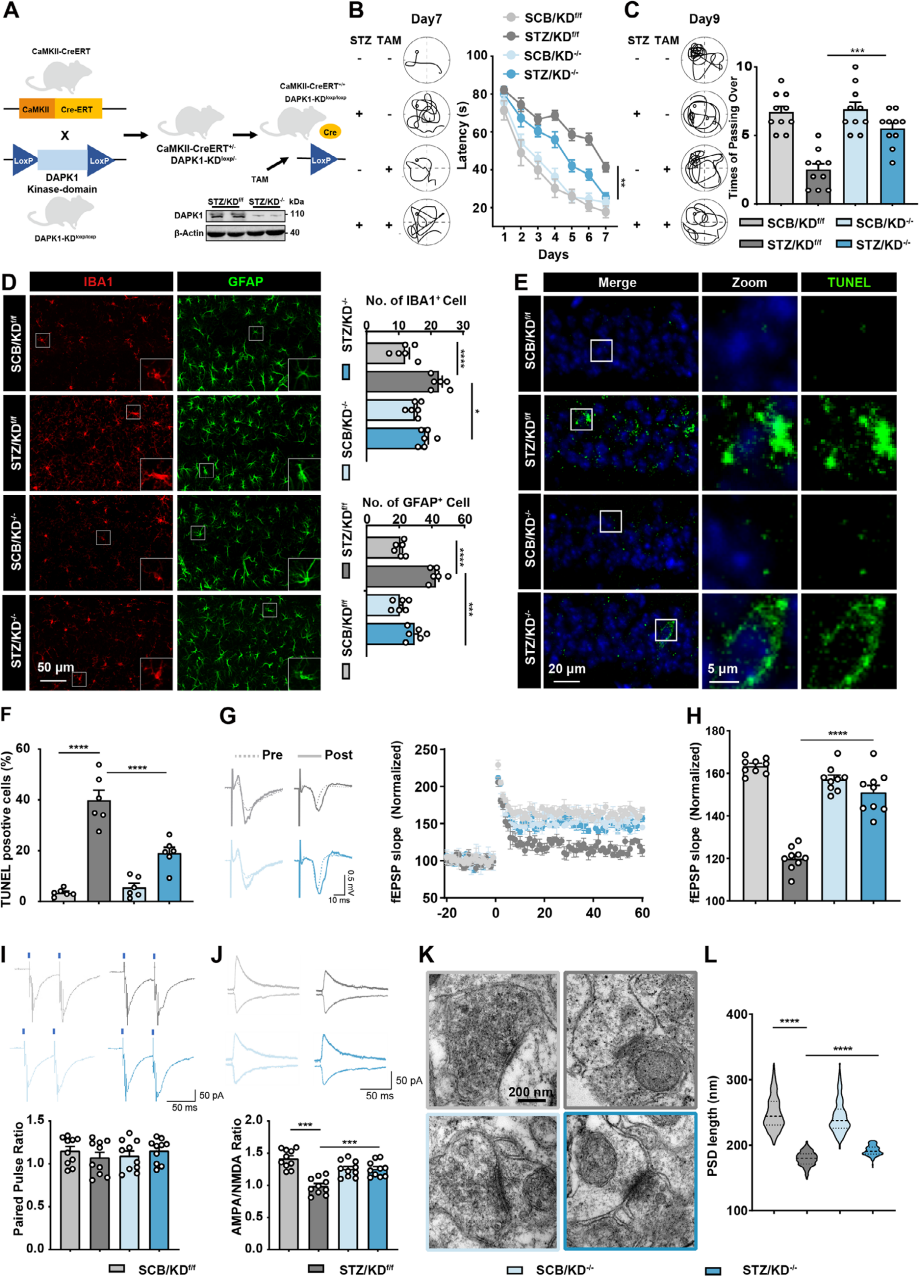
Knockdown hippocampus DAPK1 in excitatory neurons rescued diabetic encephalopathy in diabetes. A) Schematic of the strategy for conditional DAPK1 kinase domain knockout in hippocampal excitatory neurons using CaMKII‐CreERT and tamoxifen (TAM) induction in diabetic mice. Western blot analysis of DAPK1 and β‐actin protein expression in the hippocampus of STZ/KD^f/f^ and STZ/KD^−/−^ groups. *n* = 2 per group. B) Representative path traces to the hidden platform on day 7 and latency over 7 days in different groups. *n* = 10 per group. C) Swimming paths on day 9 and number of crossing the platform region on day 9 in the MWM. *n* = 10 per group. D) Immunofluorescence staining of IBA1 (red) and GFAP (green) in the hippocampus, showing changes in microglial and astrocytic activation across groups. *n* = 6 slices from three mice per group. E) TUNEL staining of hippocampal sections for apoptosis detection, with DAPI counterstaining; apoptotic cells appear green in images. F) Quantification of TUNEL‐positive cells in the hippocampus, showing reduced apoptosis in DAPK1^−/−^ mice. *n* = 6 slices from three mice per group. G) Electrophysiological recordings illustrating normalized field excitatory postsynaptic potential (fEPSP) slopes in CA3–CA1 synapses, with reduced long‐term potentiation (LTP) impairment in DAPK1^−/−^ mice. H) Summary of fEPSP slope changes among groups, highlighting improved synaptic function in DAPK1^−/−^ mice. *n* = 9 cells from three mice per group. I) Representative traces (up) and quantification (down) of paired‐pulse facilitation (PPF) recorded from hippocampal slices. *n* = 10 cells from three mice per group. J) Representative traces (up) and quantification (down) of AMPA/NMDA current ratio measured in CA1 neurons. *n* = 10 cells from three mice per group. K) Representative transmission electron microscopy (TEM) images showing hippocampal synapses in the stratum radiatum. L) Quantification of postsynaptic density (PSD) length from TEM images. *n* = 130–150 synapses from 3 to 5 mice per group. Data are shown as mean ± S.E.M., and statistical analysis was performed using one‐way or two‐way analysis of variance (ANOVA). * *p* < 0.05, ** *p* < 0.01, *** *p* < 0.001, and **** *p* < 0.0001.

Given that the elevation of DAPK1 is primarily located in excitatory neurons of the hippocampus, we next asked whether knocking down DAPK1 kinase domain in these neurons could rescue the memory impairments observed in diabetic models. To this end, we generated conditional DAPK1 kinase domain knockout mice by crossing Calcium/Calmodulin‐dependent kinase II‐driven tamoxifen‐inducible Cre recombinase (CaMKII Cre‐ERT) mice with DAPK1 kinase domain floxed mice, and we named as DAPK1‐KD^loxp/loxp^ mice. After intraperitoneal injection of tamoxifen (TAM), DAPK1 kinase domain was conditionally knocked out in CaMKII neurons, referred to as “DAPK1‐KD^−/−^” (abbreviated as “KD^−/−^”) (**Figure** **2**A). This strategy allowed us to specifically reduce DAPK1 expression in the excitatory neurons. Then, we administered STZ intraperitoneally (i.p.) to 2 month old DAPK1‐KD^−/−^mice resulting in STZ/KD^−/−^mice, and conducted a series of behavioral tests to assess the learning and memory abilities of mice 4 months later. In the MWM, compared with the STZ/KD^loxp/loxp^ group (41.4 ± 2.47 s (latency), 2.50 ± 0.43 (crossing times)), the latency of STZ/KD^−/−^ mice after knocking out DAPK1 in excitatory neurons was significantly shortened on the 7th day of training (25.42 ± 2.38 s), and the number of crossing the target area on the 9th day increased (5.50 ± 0.40) (Figure 2B,C). In the Y maze test, STZ/KD^−/−^ showed a 2.5‐fold increase in the number of spontaneous alternations compared to that of age‐matched STZ/KD^loxp/loxp^ mice (Figure S3A, Supporting Information), indicating a rescue of the hippocampus‐dependent spatial memory. Besides, we found that the discrimination index was increased in the STZ/KD^−/−^ group (33.96 ± 2.46%) compared with STZ/KD^loxp/loxp^ group (24.13 ± 3.433%) in the where–which test (Figure S3B, Supporting Information). STZ/KD^−/−^ mice (68.54 ± 2.83 s) spent ≈1.5 times as much time exploring the novel object compared to the control group (42.00 ± 2.96 s) (Figure S3C, Supporting Information). And we found that STZ/KD^−/−^ mice showed significant increased freezing behaviors in the contextual and cued test in the FCT, suggesting the deletion of DAPK1 improved contextual memory (Figure S3D, Supporting Information). These results suggest that knocking out DAPK1 in excitatory neurons alleviates the cognitive deficits observed in diabetic mouse models. And we found that microglial activation and astrocytic proliferation were significantly reduced in the STZ/KD^−/−^ group compared to controls (Figure 2D). TUNEL staining revealed a lower percentage of apoptotic cells, indicating reduced neuronal death in the STZ/KD^−/−^ group (Figure 2E,F). Additionally, the fEPSP slope was significantly improved in these mice, suggesting restored synaptic function (Figure 2G,H). These results demonstrate that DAPK1 knockdown alleviates neuroinflammation, reduces apoptosis, and mitigates synaptic disorder in diabetic mice.

To further characterize synaptic alterations beyond long‐term potentiation (LTP), we performed additional electrophysiological recordings in hippocampal CA1 pyramidal neurons. Paired‐pulse facilitation (PPF), which reflects presynaptic release probability, showed no significant difference among groups (Figure 2I), suggesting that presynaptic function was largely preserved. In contrast, the α‐amino‐3‐hydroxy‐5‐methyl‐4‐isoxazolepropionic acid (AMPA)/ N‐methyl‐D‐aspartate (NMDA) current ratio, an indicator of postsynaptic receptor composition and excitatory synaptic efficacy, was significantly decreased in diabetic mice (STZ/KD^f/f^, 0.988 ± 0.041), and this reduction was ameliorated in STZ/KD^−/−^ mice (1.253 ± 0.038) (Figure 2J).

To support these functional results, we observed hippocampal synaptic ultrastructure (Figure 2K) using transmission electron microscopy (TEM). STZ/KD^f/f^ mice exhibited shorter postsynaptic density (PSD) lengths (249.28 ± 2.17) (Figure 2L) and disorganized synaptic morphology, indicating impaired structural integrity of excitatory synapses. These abnormalities were partially rescued by DAPK1 deletion in STZ/KD^−/−^ (243.48 ± 2.15) (Figure 2L). Together, these results highlight a primarily postsynaptic deficit under diabetic conditions and support a protective role of DAPK1 deletion in maintaining synaptic structure and function.

### 2.3 The Elevation of DAPK1 Was Caused by the Suppression of miR‐216a‐5p

**Figure 3:**
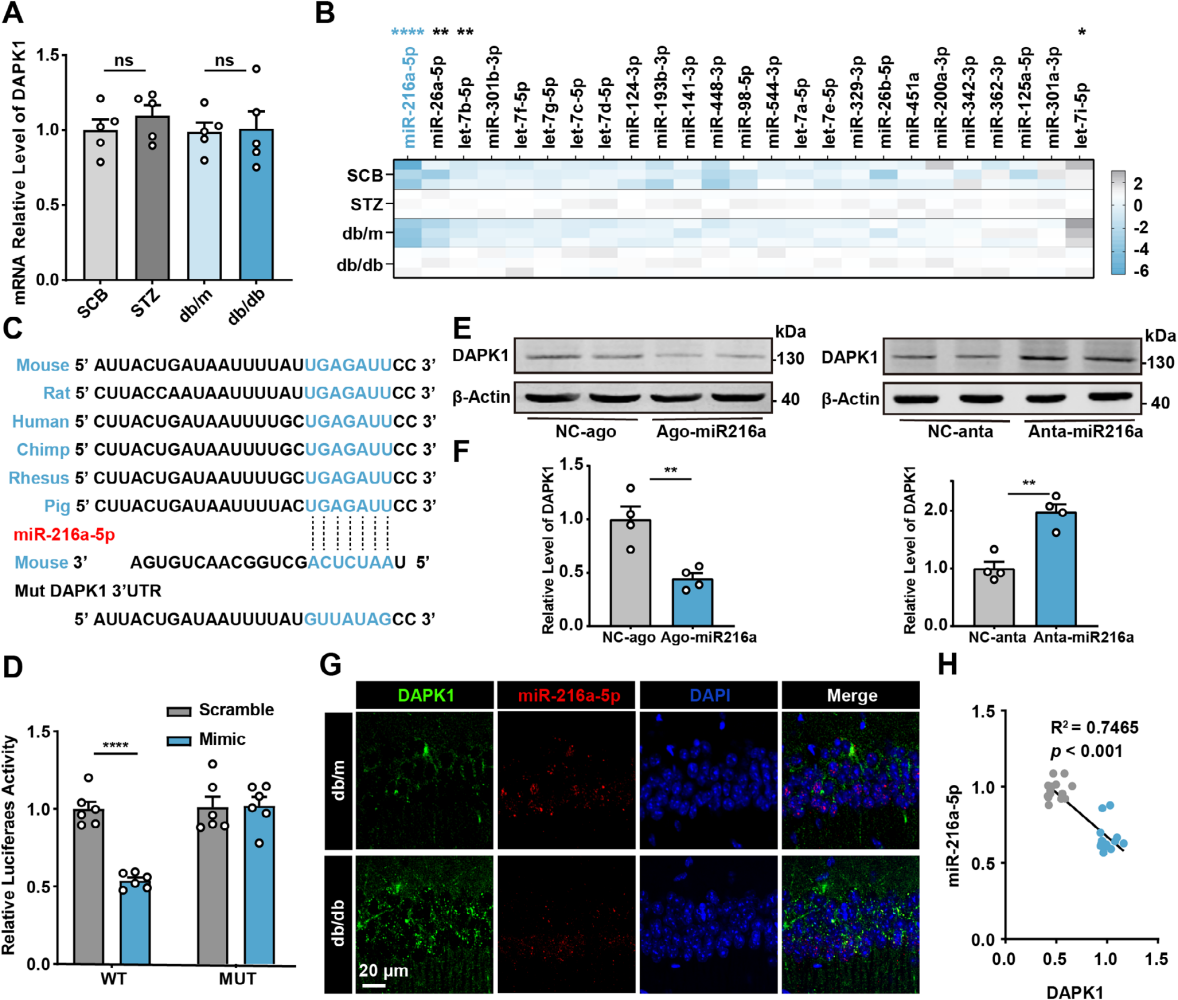
The elevation of DAPK1 is driven by suppression of miR‐216a‐5p. A) Relative mRNA levels of DAPK1 in hippocampus of control (SCB and db/m) and diabetic (STZ and db/db) mice, showing no significant differences across groups. *n* = 5. B) Heatmap of miRNA expression profiles in different groups. C) Diagram to display the conserved binding site in DAPK1 3′UTR to the miR‐216a‐5p. The mutant sequence in 3′UTR of DAPK1 for luciferase analysis was provided at the bottom. D) Luciferase reporter assay of wild‐type (WT) and mutant (MUT) DAPK1 3′UTR constructs in cells transfected with miR‐216a‐5p mimic or scramble control. *n* = 6 per group. E,F) N2a cells were transfected with miR‐216a‐5p agomir or antagomir. E) The cell lysates were collected, and the protein levels of DAPK1 were then detected by western blot (WB) after 48 h, F) with quantification. *n* = 4 per group. G,H) Immunofluorescent staining showing DAPK1 (green) and miR‐216a‐5p (red) in the hippocampus of db/m and db/db mice, with DAPI counterstaining (blue). Correlation analysis between miR‐216a‐5p and DAPK1 levels, showing a significant negative relationship (*R* ^2^ = 0.7465, *p* < 0.001). *n* = 15 slices per group from five mice. Data are presented as mean ± S.E.M., and statistical analysis was performed using an unpaired Student's *t*‐test or linear regression. * *p* < 0.05, ** *p* < 0.01, and **** *p* <0.0001.

To explore the regulatory mechanisms behind increased DAPK1 expression in diabetic mice, we measured DAPK1 message RNA (mRNA) levels and found no significant difference between diabetic and control mice (**Figure** **3**A). In organisms, miRNAs can typically bind to target mRNAs to inhibit their translation or affect mRNA stability, leading to degradation, thereby achieving the purpose of gene expression regulation.^[^ ^67^ ^]^ Therefore, we aim to identify the miRNA molecules that regulate DAPK1 mRNA. By using online prediction tools (RNA22, miRNA.org, TargetScan, RNAInter), we identified miRNAs that were consistently present across all four databases. We selected 25 miRNAs shared among these databases for further investigation (Figure S4, Supporting Information). Next, we performed real‐time quantitative polymerase chain reaction (RT‐qPCR) to analysis miRNA, revealing that the hippocampal levels of miR‐216a‐5p were significantly reduced in diabetic mice (STZ and db/db group) compared to controls (SCB and db/m group) (Figure 3B), suggesting that miR‐216a‐5p showed the most significant differential change between the two diabetic models. We identified a conserved miR‐216a‐5p binding site in the 3' untranslated region (3′UTR) region of DAPK1 mRNA across multiple species (Figure 3C). To validate this interaction, we conducted a luciferase reporter assay using constructs containing either the wild‐type (WT) or mutated (MUT) DAPK1 3′UTR. Co‐transfection with miR‐216a‐5p mimics significantly reduced luciferase activity in cells expressing the WT but not the MUT construct, confirming the direct binding of miR‐216a‐5p to the DAPK1 3′UTR (Figure 3D). Additionally, overexpression of miR‐216a‐5p (Ago‐miR216a) significantly reduced DAPK1 protein levels, while inhibition of miR‐216a‐5p (Anta‐miR216a) increased DAPK1 expression (Figure 3E,F). These findings confirm that miR‐216a‐5p negatively regulates DAPK1 expression.

Immunofluorescence staining further demonstrated an inverse correlation between miR‐216a‐5p and DAPK1 in the hippocampus of db/db mice, with regions showing decreased miR‐216a‐5p exhibiting elevated DAPK1 levels (Figure 3G). Pearson correlation analysis confirmed a negative relationship between DAPK1 and miR‐216a‐5p expressions (Figure 3H). These results suggest that the increased DAPK1 expression in diabetic mice is at least partly due to the suppression of miR‐216a‐5p, which acts as a post‐transcriptional regulator of DAPK1.

### 2.4 Loss of Netrin‐1 Mediated the Toxic Effects of DAPK1 Upregulation in Diabetic Mice

**Figure 4:**
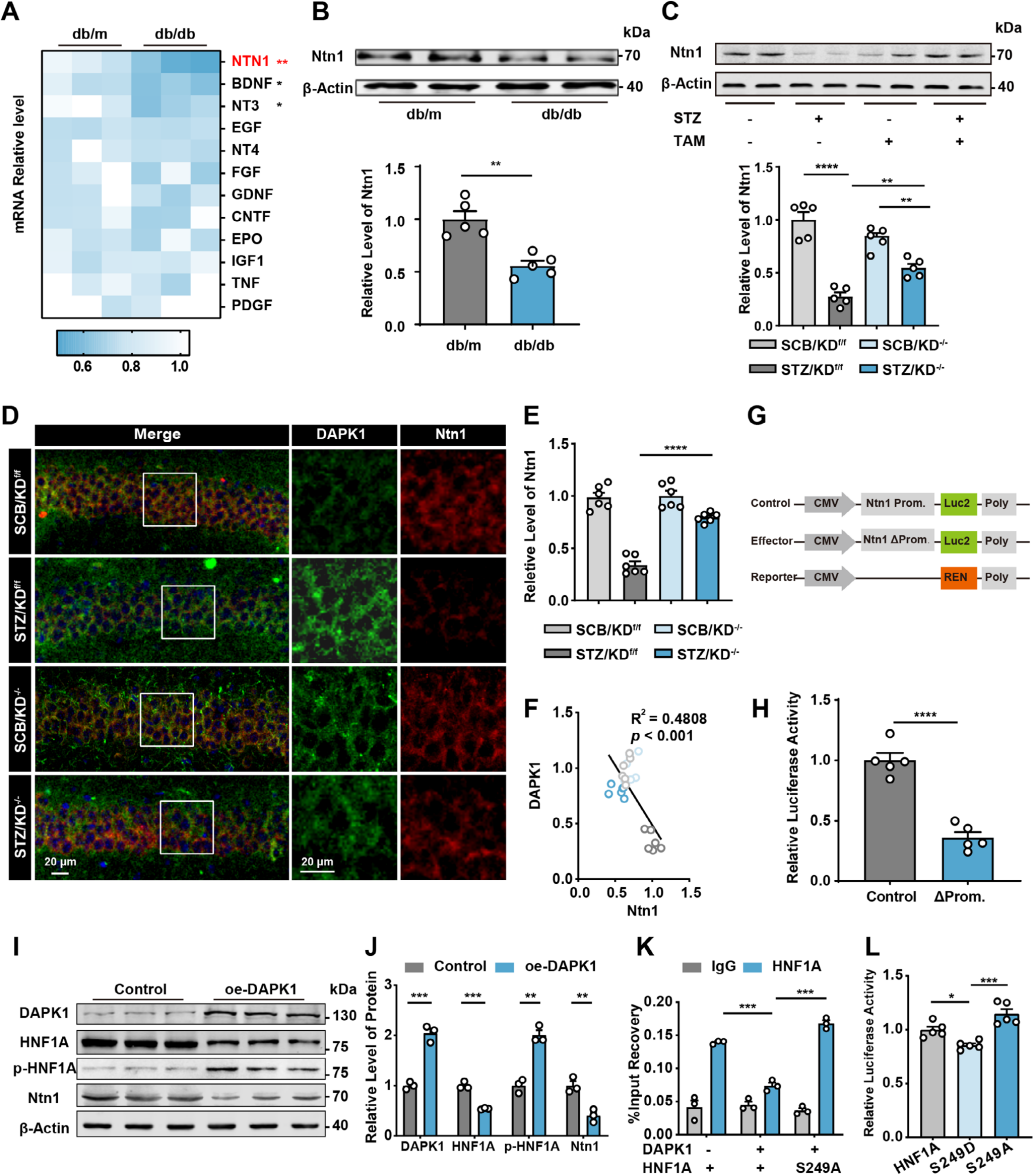
Knockdown of DAPK1 in excitatory neurons restores Netrin‐1 (Ntn1) expression. A) Heatmap showing the relative mRNA levels of various neurotrophic factors, including Ntn1, in db/db mice compared to controls. *n* = 3 per group. B) Western blot analysis of Ntn1 protein levels in the hippocampus of db/m and db/db mice, with quantification showing a significant reduction of Ntn1 in db/db mice. *n* = 5 per group. C) Western blot analysis of Ntn1 in DAPK1‐KD^+/−^ and DAPK1‐KD^−/−^ diabetic mice treated with tamoxifen (TAM), with quantification indicating restoration of Ntn1 levels following DAPK1 knockdown. *n* = 5 per group. D) Immunofluorescent staining for DAPK1 (green) and Ntn1 (red) in the hippocampus of different groups, with merged images showing colocalization. E) Quantitative analysis of Ntn1 levels in different group mice, demonstrating increased Ntn1 expression following DAPK1 kinase domain knockdown. *n* = 6 slices per group from three mice. F) Correlation analysis between the relative protein levels of DAPK1 and Ntn1 (*R* ^2^ = 0.4808, *p* < 0.001). *n* = 6 per group. G) Schematic of the dual‐luciferase reporter assay construct used to assess Ntn1 promoter activity in response to DAPK1 kinase domain knockdown. H) Relative luciferase activity in cells after cotransfection with the reporter vector and the control or effector vector for 48 h. I) Western blot analysis of HNF1A and Ntn1 levels in control and DAPK1‐overexpressing (oe‐DAPK1) cells, with J) quantification showing a decrease in HNF1A and Ntn1 levels upon DAPK1 overexpression. *n* = 3 per group. K) Chromatin immunoprecipitation (ChIP) assay was performed to assess the binding of HNF1A to the Ntn1 promoter in HEK293T cells transfected with either DAPK1 vector, wild‐type HNF1A, or phosphorylation‐deficient mutant HNF1A‐S249A. Immunoprecipitated DNA was quantified using qPCR targeting the Ntn1 promoter region. *n* = 3 per group. L) Luciferase reporter assay showing the transcriptional activity of wild‐type HNF1A and its phosphorylation mutants (S249A and S249D) on the Ntn1 promoter. HEK293T cells were co‐transfected with the Ntn1 promoter‐luciferase construct and the indicated HNF1A variants. Luciferase activity was measured 24 post‐transfection and normalized to Renilla luciferase. *n* = 5 per group. Data are presented as mean ± S.E.M., and statistical analysis was performed using an unpaired Student's *t*‐test or one‐way or two‐way analysis of variance (ANOVA), unless otherwise specified. * *p* < 0.05, ** *p* < 0.01, *** *p* < 0.001, and **** *p* < 0.0001.

Previous studies had revealed that the growth factors and cytokines played an important role in maintaining the survival of neurons and normal synaptic plasticity; we then measured various growth factors and cytokines in the hippocampus of diabetic mice. Among these, Ntn1 exhibited the most pronounced reduction in mRNA levels in db/db mice compared to controls (**Figure** **4**A). Ntn1 is a laminin‐related secretory protein that guides the direction of nerve growth by binding to its receptor. In addition to its axon guidance function, it can also promote early synapse formation.^[^ ^68^ ^]^ Western blot analysis confirmed a significant decrease in Ntn1 protein levels in db/db mice (Figure 4B). Furthermore, knockout of DAPK1 in diabetic mice (STZ/KD^−/−^) restored Ntn1 levels comparable to controls (Figure 4C), suggesting that DAPK1 negatively regulates Ntn1 expression. Immunofluorescence analysis showed that in mice treated with STZ, DAPK1 was highly expressed in neurons, while Ntn1 was notably decreased. Notably, conditional knockout of DAPK1 in excitatory neurons restored the expression of Ntn1 to baseline levels, suggesting that DAPK1 regulates Ntn1 expression (Figure 4D,E). Correlation analysis revealed a strong negative correlation between DAPK1 and Ntn1 levels (Figure 4F), further indicating a regulatory relationship between these molecules.

Previous studies have shown that DAPK1 plays a role in modulating neuronal death through various mechanisms,^[^ ^40^ ^]^ including interactions with NMDA receptors^[^ ^69^ ^]^ and extracellular regulated protein kinases (ERK) signaling pathways,^[^ ^70^, ^71^ ^]^ which can impact synaptic function and cell survival.^[^ ^72^ ^]^ Ntn1 is known to guide axon growth and synaptic formation by interacting with its receptors deleted in colorectal carcinoma (DCC) and uncoordinated‐5 homologs (UNC5), depending on the cellular context and receptor composition.^[^ ^73^ ^]^ Thus, it is plausible that DAPK1, by modulating key signaling pathways, may influence Ntn1‐mediated neuronal guidance and synaptic stability.

To further clarify the specific mechanism of Ntn1 downregulation in diabetic mouse models, we analyzed potential transcription factors that may bind to Ntn1 using the prediction website (jingle.shinyapps.io/TF_Target_Finder/) by integrating multiple databases, including Knock TF (http://www.licpathway.net/KnockTF/index.html), ENCODE (https://www.encodeproject.org/), GTRD (https://gtrd.biouml.org/), and FIMO‐JASPAR (https://meme‐suite.org/meme/doc/fimo.html, https://jaspar.elixir.no/). By merging the analysis outputs from the above databases, we noticed that hepatocyte nuclear factor 1 homeobox A (HNF1A) and tumor protein P53 (TP53) as potential regulators for Ntn1 transcription (Figure S5A, Supporting Information). Previous studies have shown that HNF1A is widely expressed in pancreatic β‐cells, liver, intestines, and other organs. Defects in this gene are a cause of maturity‐onset diabetes of the young type 3 (MODY3).^[^ ^74^, ^75^ ^]^

Furthermore, to confirm that HNF1A acts as a transcription factor by binding to the promoter or enhancer region of Ntn1, we used the JASPAR database (https://jaspar.elixir.no/) to predict potential binding sites of HNF1A within the Ntn1 promoter region (Figure S5B, Supporting Information). We then selected a promoter region of Ntn1 containing the predicted binding site and mutated the sixth base “T” to “C.” This mutated promoter sequence was inserted into a reporter plasmid. The reporter plasmid was transfected into HEK293 cells, and 48 h later, cell lysates were collected for dual‐luciferase activity detection. The results showed that the luciferase activity ratio in the mutated reporter group significantly decreased compared to the control group (Figure 4G,H), indicating that HNF1A activates Ntn1 transcription by binding to the Ntn1 promoter. We then queried how DAPK1 upregulation could regulate the Ntn1 transcription. To this end, we overexpressed DAPK1 in N2a cells and observed a significant increase in the phosphorylation level of HNF1A, ≈1.9‐fold, along with a decrease in Ntn1 protein expression, as demonstrated by western blot analysis and statistical evaluation (Figure 4I,J). These results indicate that the DAPK1‐mediated downregulation of Ntn1 occurs, at least in part, through transcriptional regulation. These findings collectively indicate that DAPK1 potentially negatively regulates Ntn1 expression in the diabetic brain. Besides, we observed that DAPK1 overexpression markedly increased the phosphorylation level of HNF1A. We therefore hypothesized that this post‐translational modification may be involved in the downregulation of Ntn1 expression. To test this, we performed chromatin immunoprecipitation (ChIP)‐qPCR to examine whether HNF1A phosphorylation affects its binding to the Ntn1 promoter. The results showed that DAPK1 overexpression significantly reduced HNF1A occupancy at the Ntn1 promoter, while expression of the dephospho‐mimetic mutant HNF1A–S249A slightly enhanced promoter binding (Figure 4K), indicating that phosphorylation of HNF1A reduces its DNA‐binding capacity.

To further assess the effect of phosphorylation on transcriptional regulation, we conducted dual‐luciferase reporter assays. Consistently, the transcriptional activity of HNF1A on the Ntn1 promoter was significantly diminished in the phospho‐mimetic S249D mutant (0.856 ± 0.017), whereas the non‐dephospho‐mimetic S249A mutant retained a relatively higher level of transcriptional activity (1.146 ± 0.046) (Figure 4L). Together, these findings suggest that DAPK1 may suppress Ntn1 transcription by promoting HNF1A phosphorylation, thereby impairing its DNA‐binding and transcriptional activities.

To further validate the functional role of the miR‐216a‐5p/DAPK1/Ntn1 signaling axis, we performed rescue experiments in primary hippocampal neurons. As expected, knockdown of miR‐216a‐5p significantly decreased dendritic spine density and increased apoptosis, as evidenced by reduced co‐localization of PSD95 and GluA1 (5.467 ± 0.496) (Figure S6A,B, Supporting Information) and elevated TUNEL‐positive cell numbers (63.167 ± 2.023) (Figure S6C,D, Supporting Information). Remarkably, co‐expression of Ntn1 in miR‐216a‐5p knockdown neurons restored dendritic spine density (10.00 ± 0.640) and markedly reduced apoptosis (50.333 ± 2.275), suggesting that Ntn1 functions downstream of miR‐216a‐5p to regulate synaptic integrity and neuronal survival.

These findings provide strong functional evidence supporting the hypothesis that miR‐216a‐5p modulates synaptic plasticity and neuronal viability via the DAPK1/HNF1A/Ntn1 pathway, and that Ntn1 overexpression is sufficient to rescue miR‐216a‐5p deficiency‐induced neurodegenerative phenotypes.

### 2.5 Intranasal Administration of Recombinant Ntn1 Slowed Down Diabetic Encephalopathy in Diabetic Mice

**Figure 5:**
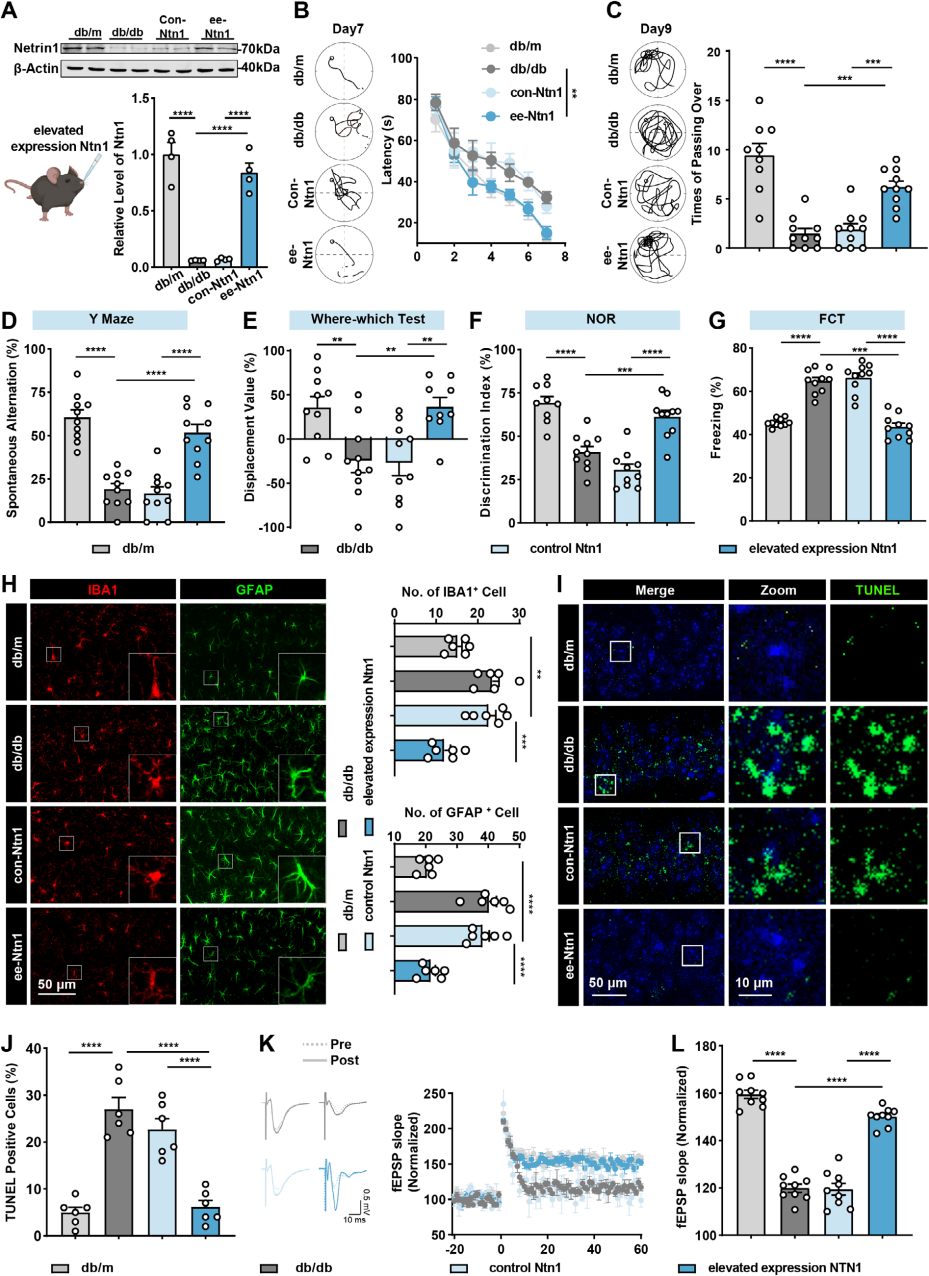
Intranasal administration of recombinant Ntn1 slowed down diabetic encephalopathy in diabetic mice. A) Western blot analysis and quantification of Ntn1 protein expression relative to β‐actin in the hippocampus of db/m, db/db, control‐Ntn1 (con‐Ntn1), and elevated expression Ntn1 (ee‐Ntn1) mice. *n* = 4 per group. B) The representative swimming path on day 7 (left) and latency to reach a platform from day 1 to day 7 (right) in the Morris water maze tasks of different groups. *n* = 10 per group. C) The representative swimming path and the number of crossings the platform region on day 9 in the Morris water maze tasks of different groups. *n* = 10 per group. D–G) Behavioral performance in D) Y maze, E) WWT, F) NOR, and G) FCT tests following Ntn1 overexpression showed significant improvements, indicating enhanced spatial memory and cognitive flexibility in mice. *n* = 10 per group. H) Representative images and qualification of microglia (IBA1, red) and astrocytic (GFAP, green) activation in the hippocampus of the ee‐Ntn1‐treated mice compared to controls. *n* = 6 slices from three mice per group. I,J) Representative images and quantification of apoptotic cells, as indicated by TUNEL‐positive staining (green). Nuclei were stained with DAPI (blue). The regions marked with white squares in the CA1 area are shown at higher magnification on the right. The percentage of apoptotic cells was calculated with the number of TUNEL‐positive cells divided by the total number of DAPI‐labeled cells in the same microscopic field. *n* = 6 slices from three mice per group. K,L) Electrophysiological recordings of K) fEPSP slope and L) quantitative analysis to evaluate the LTP at the CA3‐CA1 synapse. *n* = 9 slices from three mice per group. Data are presented as mean ± S.E.M., and statistical analysis was performed using an unpaired Student's *t*‐test or one‐way or two‐way analysis of variance (ANOVA), unless otherwise specified. ** *p* < 0.01, *** *p* < 0.001, and **** *p* < 0.0001.

We then asked whether supplement of exogenous Ntn1 could slow down cognitive impairment and encephalopathy in diabetic mice. We then delivered the recombinant Ntn1 by intranasal administration and found it effectively increased the level of Ntn1 in the hippocampus of STZ mice (**Figure** **5**A). To investigate the impact of exogenous Ntn1 supplementation on cognitive behavior, we next assessed the changes in cognitive function in diabetic mice following Ntn1 treatment. In the MWM, Ntn1‐treated mice (elevated expression Ntn1, ee‐Ntn1) showed shorter latencies (16.54 ± 1.99 s) from day 3 during the learning phase and less across time in the platform area (6.20 ± 0.59) during the probe trial compared those of control mice (28.08 ± 3.50 s (latency), 1.90 ± 0.59 (across times)) (Figure 5B,C). Additionally, we found that compared to the control group, the ee‐Ntn1 mice showed increased spontaneous alternation (69.22 ± 3.69%) in the Y maze (Figure 5D), an elevated discrimination index (25.32 ± 13.95%) in the WWT test (Figure 5E), and a higher discrimination index (51.69 ± 4.80%) when exploring novel objects in the NOR test (Figure 5F). In the FCT, ee‐Ntn1 mice exhibited reduced freezing behavior in the contextual (43.54 ± 1.73 s) (Figure 5G). Also, ee‐Ntn1 reduced neuronal apoptosis and glial activation in the hippocampus, relieving diabetes‐induced pathological changes (Figure 5H–J). And electrophysiological recording in the CA1 region of the hippocampus suggested that the reduction of LTP was restored in ee‐Ntn1 mice, but not in control mice (Figure 5K,L). These results suggest that overexpression of Ntn1 can rescue diabetes‐related cognitive impairments and pathological changes in mice.

### 2.6 Silencing of Netrin‐1 Induced the Diabetic‐Like Memory Impairments and Diabetic Encephalopathy

We then queried whether the loss of Ntn1 is responsible for the diabetic encephalopathy. We infused of a specific short hairpin RNA (shRNA) that targeting Ntn1 into the hippocampus of wild‐type mice, and found that it significantly reduced the expression of Ntn1 (Figure S7A, Supporting Information). Importantly, Ntn1 knockdown impaired learning and memory, evidenced by longer latencies (34.59 ± 3.38 s) and reduced platform crossings (1.60 ± 0.37) in the Morris water maze (Figure S7B,C, Supporting Information), lower discrimination indices (6.76 ± 2.29%, 0.37 ± 2.34%) in both the Y maze (Figure S7D, Supporting Information) and WWT (Figure S7E, Supporting Information), decreased recognition ability (38.32 ± 2.13%) in the NOR test (Figure S7F, Supporting Information), and increased freezing behavior (63.07 ± 1.74 s) in the FCT (Figure S7G, Supporting Information). Additionally, Ntn1 silencing led to neuronal loss and increased glial activation in the hippocampus (Figure S7H–J, Supporting Information). Electrophysiological recordings confirmed that LTP at CA3–CA1 synapses was impaired in Ntn1 knockdown mice (Figure S7K,L, Supporting Information). These findings further demonstrated the critical role of Ntn1 in regulating cognitive function and diabetes related pathological changes.

## 3 Discussion

In this study, the diabetic model was established by using db/db mice and STZ injection in mice. The spatial learning and memory function was assessed via MWM, Y maze, WWT, FCT, and NOR, which are the widely used cognitive‐related behavioral experiments. We found that the performances were worse across a variety of behavioral tests in db/db and STZ injection mice. After transgenic knocking out DAPK1‐KD in CaMKII‐positive excitatory neurons or intranasal administration of recombinant Ntn1, the performances were reduced in STZ/DAPK1‐KD^−/−^ or db/db mice. Moreover, intranasal administration of recombinant Ntn1 also improved cognitive function in db/db mice. Based on our data, we believed that both DAPK1‐KD knockout and invasive administration Ntn1 recombinant proteins can improve cognitive impairment in diabetic mice.

Previous studies have shown that DAPK1 is primarily expressed in the brain.^[^ ^76^ ^]^ In addition, there was a gradual decline in global expression of DAPK1 mRNA in the brain in the postnatal stage, and expression was limited to hippocampal neurons, suggesting that DAPK1 may be involved in neuronal function in the hippocampus.^[^ ^77^ ^]^ Our previous studies have shown that DAPK1 interacts with excitatory glutamate receptor NR2B subunit at extrasynaptic sites physically and functionally and become activated in response to stroke damage.^[^ ^41^ ^]^ Here, we demonstrated that there was a significant increase of DAPK1 in the hippocampus of diabetic mice, and it may lead to a deterioration of synaptic plasticity with LTP aberration. After knocking out DAPK1‐KD in diabetic mice (STZ/DAPK1‐KD^−/−^ mice) or intranasal administration of recombinant Ntn1 in diabetic mice (db/db mice), synaptic plasticity can be partially restored. In summary, diabetes mellitus may be another predisposing factor for DAPK1 activation and impaired synaptic function similar to stroke.

Glucose is the main circulating carbohydrate, and its metabolism must be strictly regulated by the body to ensure balanced energy distribution. Previous studies have shown that the expression and function of miRNAs can participate in the regulation of glucose metabolism. miRNAs can regulate insulin synthesis and secretion by modulating key enzymes involved in glucose catabolism and ATP production in pancreatic β‐cells, thereby influencing glucose metabolism.^[^ ^78^ ^]^ miR‐130a, miR‐130b, and miR‐152 exert negative regulation on glucokinase (GCK) and pyruvate dehydrogenase E1 alpha subunit (PDHA1).^[^ ^79^ ^]^ GCK plays a role in the first step of glycolysis. Both proteins are involved in ATP production. PDHA1 is a subunit of pyruvate dehydrogenase, which converts pyruvate produced in glycolysis in the mitochondria into acetyl‐CoA. Additionally, miRNAs also affect insulin signaling downstream responses in peripheral tissues such as the liver and muscles, regulating glucose storage and synthesis.^[^ ^80^ ^]^ In healthy mice, miR‐185‐5p can inhibit hepatic gluconeogenesis by suppressing the G6Pase axis, thereby lowering fasting blood glucose levels.^[^ ^81^ ^]^ Our findings indicate that in diabetic mice, the levels of several miRNAs in the hippocampus are reduced, with miR‐216a‐5p showing the most significant decrease, which may be related to the changes in blood glucose levels in diabetic mice.

Nerve growth factor (NGF) is a critical growth factor that promotes neuronal survival and growth.^[^ ^82^ ^]^ It plays an essential role in synaptic maturation, axonal targeting, synaptic plasticity, and neuronal development.^[^ ^83^, ^84^, ^85^, ^86^ ^]^ In diabetic mice, reduced NGF expression has been observed in neural and peripheral tissues during the onset of neuropathy.^[^ ^87^, ^88^, ^89^ ^]^ Furthermore, studies have shown that NGF levels are significantly decreased in the blood of diabetic patients with peripheral neuropathy.^[^ ^90^ ^]^ Diabetes also leads to the reduction of angiogenic mediators such as NGF, insulin‐like growth factor, and vascular endothelial growth factor, impairing neural nutrition and exacerbating neuropathy.^[^ ^91^ ^]^ Previous research has indicated that DAPK1 mediates neuronal loss and exacerbates brain tissue damage through the ERK/cAMP response element‐binding protein (CREB)/BDNF signaling pathway, leading to cognitive dysfunction in PSD rats.^[^ ^71^ ^]^ Given these findings, it is evident that alterations in neurotrophic factor expression are closely associated with neuronal damage and cognitive decline in diabetes.

To further investigate the changes in neurotrophic factor expression in the brains of diabetic mice, we screened several common neurotrophic factors, including BDNF, NT3, NT4, fibroblast growth factor (FGF), glial cell line‐derived neurotrophic factor (GDNF), and Ntn1. Among these factors, we found a significant reduction in Ntn1 expression in the hippocampus of diabetic mice. This suggests that Ntn1 may play a critical role in the cognitive impairments associated with diabetes.

As a classic neural guidance molecule, Ntn1 is not only crucial during neural development but also exerts protective effects on neuronal function by regulating neuroinflammation and synaptic plasticity.^[^ ^92^, ^93^ ^]^ The observed reduction in Ntn1 highlights its potential involvement in the pathophysiology of diabetes‐related cognitive dysfunction, warranting further investigation into its regulatory mechanisms and therapeutic potential.

The pathophysiology of learning and memory impairment caused by diabetes is multifactorial, involving neuroinflammation, oxidative stress, nerve damage, and microvascular dysfunction.^[^ ^94^ ^]^ These mechanisms collectively contribute to hippocampal pathology, leading to cognitive deficits. Previous studies have demonstrated that hippocampal neuronal apoptosis is induced in diabetic animal models, accompanied by impairments in spatial memory and cognition.^[^ ^17^, ^33^ ^]^ Moreover, diabetes is known to exacerbate microglial activation and the production of inflammatory cytokines. The activation of glial cells, including astrocytes and microglia, has been shown to promote neuronal cell death and result in cognitive deficits.^[^ ^95^, ^96^ ^]^ Synaptic plasticity, a critical determinant of learning and memory, is also compromised in diabetes.^[^ ^97^ ^]^ The mechanistic target of rapamycin (mTOR)/NF‐κB signaling pathway has been identified as a key regulator in this process. Inhibition of this pathway has been reported to reduce inflammation, increase synaptic protein expression, and improve ultrastructural synaptic plasticity in the hippocampus of diabetic mice.^[^ ^98^, ^99^ ^]^ These findings emphasize the importance of targeting diabetic encephalopathy, including neuroinflammation, neuronal cell death, and synaptic dysfunction, in alleviating cognitive deficits associated with diabetes.

In conclusion, our study reveals a novel miR‐216a‐5p/DAPK1/HNF1A/Ntn1 signaling axis that critically contributes to diabetes‐associated cognitive impairment. Diabetic mice exhibited pronounced deficits in learning and memory, accompanied by reduced synaptic dysfunction, increased neuronal apoptosis, and downregulation of Ntn1 expression in the hippocampus. Mechanistic investigations show that miR‐216a‐5p downregulation under diabetic conditions leads to DAPK1 upregulation, which suppresses HNF1A‐mediated transcription of Ntn1. Functional rescue experiments in both primary neurons and diabetic mice confirm that overexpression of Ntn1 significantly restores synaptic structure and reduces neuronal apoptosis, ultimately ameliorating cognitive deficits. These findings provide comprehensive in vitro and in vivo evidence that the miR‐216a‐5p/DAPK1/HNF1A/Ntn1 axis plays a central role in mediating neurodegeneration and synaptic dysfunction in diabetes. Targeting this axis may offer a promising therapeutic strategy for preventing or reversing diabetes‐related dementia. Targeting this axis may offer a promising therapeutic strategy for preventing or reversing diabetes‐related dementia. Future studies will be needed to explore whether modulation of this pathway can yield long‐term neuroprotective effects and cognitive benefits in clinical settings, and to investigate how this regulatory mechanism interacts with other metabolic or inflammatory signals in the diabetic brain.

## 4 Experimental Section

### Animals

The C57BL/6J mice (JAX: catalog no. 000664; RRID: IMSR_JAX: 000664) and CaMKIIα‐Cre mice (JAX: catalog no. 005359; RRID: IMSR_JAX:005359) were purchased from the Jackson Laboratory (Bar Harbor, ME). 2 month old male db/m and db/db mice were purchased from the Nanjing University Model Animal Research Center (Nanjing, China). The DAPK1‐kinase‐domain^loxp/loxp^ mice (abbreviated as DAPK1^loxp/loxp^ mice) were provided by Prof. Lu Youming's lab at Huazhong University of Science and Technology. The animal procedures were approved by the Animal Care and Use Committee of Tongji Medical College (Approval No. 2019‐S855).

### Generation of CaMKII Neuron‐Specific Conditional Knockout DAPK1 Kinase Domain Transgenic Mice

CaMKIIα‐CreERT2 homozygous mice were crossed with DAPK1‐kinase‐domain^loxp/loxp^ mice to produce CaMKIIα‐CreERT2^+/+^ × DAPK1‐kinase‐domain^loxp/loxp^ double homozygous mice. TAM (MCE, HY‐13757A) was administered intraperitoneally for ten consecutive days to induce Cre‐mediated recombination. Two weeks after TAM treatment, the mice with excitatory neurons in the CaMKIIα region specifically knocking out the kinase domain of DAPK1 (DAPK1‐KD^−/−^) were obtained. The control group received the same dose of corn oil (vehicle, MCE, No.8001‐30‐7) via intraperitoneal injection. They were bred in the Experimental Animal Central of Tongji Medical College, Huazhong University of Science and Technology. A 12 h light/dark cycle was maintained, and the animals were provided with ad libitum access to food and water.

### Establishment of the Diabetes Model^[^ ^100^, ^101^ ^]^

To establish the diabetes model, 8 week old male C57BL/6J mice were randomly divided into two groups: the control group (SCB) and the diabetes group (STZ). Mice in the diabetes group were first fed a high‐fat diet (HFD; XTHF60, Research Diets, USA) containing 60 kcal% fat, 20 kcal% carbohydrate, and 20 kcal% protein, while the control group received a normal control diet (NCD) containing 10 kcal% fat, 70 kcal% carbohydrate, and 20 kcal% protein. After dietary intervention, and following a 12 h fasting period, STZ (Solarbio, S8050) was dissolved in SCB (pH = 4.5) and administered i.p. at a dose of 100 mg kg^−1^. The control group received the same volume of SCB. The drug solution was prepared on ice, protected from light, and must be injected within 30 min to prevent degradation. After the injection, mice were provided with a 72 h supply of glucose water to avoid hypoglycemic death. Then random blood glucose levels were measured using a portable glucometer. Mice with blood glucose levels higher than 16.7 mmol L^−1^ were considered successfully diabetic. Behavioral testing using the water maze was then conducted to assess the cognitive abilities of the mice. The latency during the hidden platform navigation phase was significantly prolonged in the diabetic mice, confirming the success of the diabetes model.

### Intranasal Administration of Recombinant Netrin‐1^[^ ^102^, ^103^ ^]^

Intranasal administration of recombinant mouse Netrin‐1 (rNTN‐1; R&D Systems, Cat# 1109‐N1‐025/CF) was performed once daily for seven consecutive days. Mice were gently restrained in a supine position without anesthesia, and their heads were tilted back at ≈45° to facilitate nasal delivery. rNTN‐1 was dissolved in sterile phosphate‐buffered saline (PBS) at a concentration of 0.1 µg µL^−1^, and administered at a total daily dose of 1 µg per mouse, divided into two 5 µL doses (0.5 µg per dose) given in the morning and evening. Each 5 µL dose was applied alternately into the left and right nostrils using a fine pipette tip, with 2.5 µL per nostril per dosing, delivered slowly over ≈10 s. After administration, mice were held in position for ≈30 s to allow adequate absorption and minimize drainage.

### Cell Culture

Cells were maintained at 37 °C in a 5% CO_2_ humidified atmosphere. N2a and HEK293 cell lines were cultured in Dulbecco’s Modified Eagle Medium (DMEM) supplemented with 10% fetal bovine serum (FBS). Transfections of plasmids or miRNA mimics/inhibitors were performed using Lipofectamine 3000 (Lipo3000).

### Primary Cell Culture

Cultures of dissociated hippocampal neurons were prepared from embryonic E16 C57BL/6J mice as previously described.^[^ ^104^ ^]^ Cortices or hippocampi from embryos were dissected with the meninges and blood vessels removed. Minced tissues were digested in trypsin at 37 °C for 10 min. Planting media were added to terminate digestion after the tissues were fully digested. Digested tissue fragments were then filtered through a 40 µm cell strainer. Dissociated neurons were plated onto poly‐d‐lysine‐coated coverslips in a 24‐well plate containing planting medium (DMEM/F12 with 10% FBS and 1% penicillin/streptomycin) at a proper density and then incubated for 2–4 h. A maintenance medium (neurobasal medium supplemented with 2% B27, 1× GlutaMAX, and 1% penicillin/streptomycin) was then replaced after attachment, which was changed every 3 days with maintenance medium containing cytarabine (2 µm).

### RNA Isolation and RT‐qPCR^[^ ^105^ ^]^

Total RNA was extracted using TRIzol regent (Invitrogen, CA, USA) following the manufacturer's instructions. To prepare RNA for PCR analysis, a total of 1 µg RNA was transcribed into complementary DNA (cDNA) using the Hifair II1st Strand cDNA Synthesis Kit (Yeasen, 11123ES10). Realtime PCR program was performed in a Cycler (Bio‐Rad). The relative expression levels of mRNAs or miRNAs were quantified using the iTaq Universal SYBR Green Supermix (Bio‐Rad) on the real‐time PCR detection System (Applied Biosystems). The primers used to detect mRNAs and miRNAs are listed in the Supporting Information.

### Dual‐Luciferase Assay^[^ ^106^ ^]^

The 3′UTR of DAPK1 (NM_001285917.1) was cloned and inserted into psiCHECK2 vector within the Xho1 and Not1 restriction sites located downstream of the Renilla luciferase gene. Mutation of the sequence was performed using a QuickMutation Site‐Directed Mutagenesis kit (D0206S, Beyotime) to construct a control plasmid. For the promoter luciferase reporters, the plasmids containing the WT or mutant DAPK1 3′UTR were cotransfected into HEK293T cells with miR‐216a‐5p or NC‐agomirs. Cells were harvested after 48 h of transfection, and luciferase assays were performed using a dual‐luciferase reporter assay kit (Promega) according to the manufacturer's instructions. The values of Renilla activity relative to firefly activity were used for analysis.

### ChIP‐qPCR

Potential HNF1A binding sites within 2 kb upstream of the Ntn1 transcription start site (TSS) were predicted using the JASPAR database (http://jaspar.genereg.net/) under default settings. Based on the predicted motifs, specific primers were designed for subsequent ChIP‐qPCR analysis. Chromatin immunoprecipitation was performed using a commercial ChIP assay kit (#P2078, Beyotime) following the manufacturer's instructions. Briefly, cells were crosslinked with 1% formaldehyde at 37 °C for 10 min and quenched with 125 mm glycine. Cells were then lysed in sodium dodecyl sulfate (SDS) lysis buffer, and chromatin was sheared to 200–1000 bp fragments using a probe sonicator (VCX‐150, Sonics). After sonication, lysates were centrifuged at 12 000  ×  *g* for 5 min at 4 °C, and the supernatant containing soluble chromatin was collected.

Sheared chromatin was incubated overnight at 4 °C with an anti‐HNF1A antibody (#22426‐1‐AP, Proteintech) or control rabbit Immunoglobulin G (IgG). Antibody–chromatin complexes were captured using protein A‐agarose beads, followed by a series of washes with appropriate buffers. Bound chromatin was eluted from the beads, and crosslinks between DNA and proteins were reversed by incubation at 65 °C overnight. DNA was then purified using a standard purification protocol. Enriched DNA and input controls were analyzed by qPCR using specific primers targeting predicted HNF1A binding regions upstream of Ntn1.

### OGTT and ITT^[^ ^107^ ^]^

Fasting blood glucose (FBG) was measured using a handheld glucometer (Sinocare Anwen+, China). Mice were fasted overnight with free access to water. The tail tip (≈1 mm) was clipped, and the first drop of blood was discarded. After a 12 h fasting period, mice were administered a 20% glucose solution (10 mL kg^−1^ body weight) via oral gavage. Blood glucose levels were measured at 0, 15, 30, 60, 90, and 120 min. For the ITT experiment, after a 2–5 h fasting period, mice were intraperitoneally injected with insulin (0.75 U kg^−1^ body weight), and blood glucose levels were monitored at the same time points as in the OGTT

(1) $$\mathrm{HOMA}-\mathrm{IR}=\frac{\mathrm{FPG}\left(\mathrm{mmol}\;L^{-1}\right)\times \mathrm{FPI}\left(\mathrm{pmol}\;L^{-1}\right)}{22.5}$$

where HOMA‐IR is the homeostatic model assessment for insulin resistance; FPG is the fasting plasma glucose; and FPI is the fasting plasma insulin.

### Y Maze Test

The Y maze was widely used to perform a continuous spontaneous alternation test evaluate short‐term working memory. The Y maze apparatus consists of three identical arms at 120° which each was 40 cm in length and 8 cm in width, with walls that were 10 cm in height. The mice then were placed in the center of three arms and allowed to explore freely for 8 min. The number of continuous spontaneous alternating triad was recorded in 8 min which was defined as a consecutive entry to the three different arms. The percentage of spontaneous alternation was calculated as (total number of consecutive entry into three different arms)/(total number of arm entry − 2) × 100%.

### Morris Water Maze Test

The MWM test was described to examine spatial learning and memory of mice. The behavioral test was performed at a round water pool which was filled with water at a temperature of 23 ± 1 °C. The test consisted of two parts, namely navigation test and spatial probe test. In the navigation test, the circular platform which had a diameter of 10 cm was placed at the target quadrant, then the mice were trained to find the platform within a period of 60 s for five consecutive days, with four training sessions daily. On the 6th day, the mice were subjected to the spatial probe test and the mice were placed in the quadrant diagonally opposite to the target quadrant without the platform. The latency reaching the platform and the times of crossing the platform were recorded as well as the duration spent in the target quadrant.

### Where–Which Test^[^ ^108^ ^]^

The apparatus were as follows. The experiments were conducted in two open wooden boxes (50 × 50 × 40 cm), customized with different contexts by pasting paper in different patterns and colors at their bottom and walls. Two of object A were placed on the left and top of the context 1. And two of object B were placed on the right and the top of the context 2.

The procedure was as follows. Mice were placed in the experimental room 1 h before the beginning of the behavioral experiments for familiarization and then were placed in contexts 1 and 2 for free exploration for 10 min with a 1 h interval. On the second day, the mice were placed in contexts 1 and 2 for 2 min instead with the same experimental process as the first day. Context 1(2) was cleaned with 75% ethanol between each trial. During the test stage, two of object C were placed on the left and right of context 1 or 2, and the mice were randomly placed in context 1 or 2 for free exploration for 2 min. Time spent exploring the novel object within familiar location (TF) and novel object within novel location (TN) was measured. Sniffing and touching movements of mice 1 cm around the object were considered as exploration activity. The discrimination index was defined as follows: (TN −TF)/(TN+TF) × 100%. This behavior paradigm was performed as previously reported.

### Novel Object Recognition

The novel object recognition task was used to evaluate general cognitive function. This test was conducted in an open field arena of 40 cm long and 50 cm wide. In the first day, the mice were placed in this arena which was equipped with two identical objects and were allowed a free exploration lasting 10 min. After 24 h, the mice were exposed again to this testing environment, where one of above objects was replaced a novel object that can distinguish the appearance of the remaining object, and explored for 5 min. The discrimination index, which reflects the ability of learning and memory, was calculated as time difference between exploring the new object and exploring the familiar object/total time spent exploring both objects.

### Fear Conditioning Test

The fear conditioning test was applied to investigate hippocampus‐dependent memory of rodents. In the training phase, the mice received five cycles of a tone cue (10 kHz; 75 dB sound pressure level (SPL)) lasting 30 s and two foot shocks (2 s; 0.75 mA) after 180 s adaptive phase without any stimulation. In the test phase, the mice were again exposed to the same context without any stimulation and recorded the freezing times and duration. During the tone‐conditioned test, the mice returned to the chamber of which walls were decorated with different patterns and of which smell was sprayed with acetic acid. The freezing time was measured when the mice exposed to the tone lasting 30 s.

### Western Blotting^[^ ^109^ ^]^

Cells and frozen hippocampal samples were lysed in radio immuno precipitation assay (RIPA) extract buffer (Beyotime, Shanghai, China, Cat#P00138) supplemented with protease inhibitor cocktail (MedChem Express, USA, HY‐K0010), phosphatase inhibitor cocktail I (MedChem Express, USA, HY‐K0021), and phosphatase inhibitor cocktail II (MedChem Express, USA, HY‐K0022) according to the manufacturer's protocol. After boiling for 10 min, these samples were measured using the bicinchoninic acid (BCA) protein assay reagent (Thermo Fisher Scientific, IL, USA). Equal amounts of protein (30–50 µg) were loaded into each lane, separated by 10% SDS polyacrylamide gel electrophoresis and transferred to nitrocellulose (NC) membranes. After blocking with 5% nonfat milk for 30 min, the membranes were incubated with primary antibodies overnight at 4 °C to DAPK1 (rabbit, 1:1000, No.25136‐1‐AP, Proteintech), DAPK1 (mouse, 1:1000, No.67815‐1‐Ig, Proteintech), β‐actin (mouse, 1:800, No.66009‐1‐Ig, Proteintech), HNF1A polyclonal antibody (rabbit, 1:1000, No. 22426‐1‐AP, Proteintech), Phospho‐HNF1A (Ser247) Ab (rabbit, 1:1000, AF7045, Affinity), or Netrin‐1 (rabbit, 1:500, DF8579, Affinity), followed by washes with Tris‐buffered saline (PBS)‐Tween 20. Then, the membranes were incubated with antirabbit or antimouse immunoglobulin G conjugated secondary antibody IRdye 800 (1:10 000; Rockland Immunochemicals) for 1 h at room temperature. The protein bands were detected using the Odyssey Imaging System (LI‐COR, Lincoln, NE, USA).

### Immunofluorescence

Adult mice were anesthetized with an intraperitoneal injection of sodium pentobarbital (50 mg kg^−1^) and transcardially perfused with PBS, followed by 4% paraformaldehyde (PFA) in PBS. Brains were carefully dissected and postfixed in 4% PFA at 4 °C for 12–16 h. After fixation, brains were washed in PBS and cryoprotected by sequential immersion in 20% and 30% sucrose solutions at 4 °C until fully equilibrated. Coronal brain slices (30 µm thick) were prepared using a cryostat and stored in antifreeze solution at −20 °C until use. For immunofluorescence staining, each brain slice was rinsed three times with PBS and subsequently incubated with the primary antibody at 4 °C overnight. The slices were then incubated with a fluorescence‐conjugated secondary antibody at room temperature for 1 h. After thorough washing, the slices were mounted onto slides using an antifluorescent quencher containing 4′,6‐Diamidino‐2‐Phenylindole (DAPI) (Superkine) and cover‐slipped. For primary antibodies, antibodies against DAPK1 (mouse, 1:300, Proteintech, No.67815‐1‐lg), NeuN (rabbit, 1:500, Cell Signaling Technology, 94403S), CaMKII‐α(6G9) (mouse, 1:400, Cell Signaling Technology, 50049), GAD1 (mouse, 1:100, Santa Cruz, sc‐28376), IBA1 (Rat, 1:500, Abcam, Ab283319), GFAP (Rat, 1:500, Cell Signaling Technology, 3670), Ntn1(Rabbit, 1:200, Affinity, DF8579) were used. For the secondary antibody, antibodies from Thermo Fisher Scientific, including Alexa Fluor 488(1:800, A‐21247, A‐11001, and A‐21206), Alexa Fluor 546(1:800, A‐10036 and A‐10040) were used. Fluorescent images were acquired using a confocal laser scanning microscope (Zeiss LSM 800) under appropriate excitation and emission settings. All imaging parameters were kept consistent across samples to ensure comparability.

### Fluorescence In Situ Hybridization

Fluorescence in situ hybridization (FISH) was performed to detect specific RNA targets in mouse brain tissue sections. The specific FISH probes to miR‐216a‐5p were designed and synthesized by Tsingke Biotechnology. Brain sections were fixed with 4% paraformaldehyde for 20 min at room temperature, followed by permeabilization with 0.5% Triton X‐100 in PBS for 10 min. After washing with PBS, sections were prehybridized with hybridization buffer at 37 °C for 1 h. Subsequently, FISH probes were added to the sections and hybridized overnight at 37 °C in a humidified chamber. The following day, sections were washed with saline‐sodium citrate (SSC) buffer at varying stringency and mounted with an antifluorescent quencher containing DAPI. Fluorescent signals were visualized and captured using a ZEISS confocal microscope (LSM 800, Germany).

### TUNEL Apoptosis Assay

TUNEL staining was performed on mouse brain frozen sections using the TUNEL Apoptosis Detection Kit^[^ ^110^ ^]^ (Green Fluorescence, Abbkine, China) according to the manufacturer's protocol. Briefly, brain sections were fixed with 4% paraformaldehyde for 20 min at room temperature and permeabilized with 0.1% Triton X‐100 in PBS for 10 min. The sections were then incubated with TUNEL staining reagent at 37 °C for 1 h in the dark. Following incubation, the sections were washed in PBS and mounted with antifade medium containing DAPI for nuclear counterstaining. Fluorescent images were acquired using a ZEISS confocal microscope (LSM 800, Germany).

### Transmission Electron Microscopy

Hippocampal tissues were sliced into pieces of 1 mm^3^. The samples were immersed in 2.5% glutaraldehyde immediately after being isolated from the brain at 4 °C for 6 h. Then the pieces were fixed with 1% osmium tetroxide and dehydrated in graded ethanol series, and embedded in Araldite. Ultrathin sections (50 nm) were stained with 2% uranyl acetate for 15 min and 2% lead citrate for 15 min. Finally, the sections were observed under a transmission electron microscope (Hitachi HT7800). Sample preparation and imaging were performed at the Institute of Hydrobiology, Chinese Academy of Sciences.

### Electrophysiology

Parasagittal brain slices (400 µm) were prepared using standard procedures. Dissections and recordings were performed in ice‐cold artificial cerebrospinal fluid (ACSF) containing 120 mm NaCl, 3 mm KCl, 1.0 mm NaH_2_PO_4_, 1.5 mm MgSO_4_·7H_2_O, 2.5 mm CaCl_2_, 25 mm NaHCO_3_, and 20 mm glucose, continuously bubbled with carbogen (95% O_2_/5% CO_2_) to maintain pH and oxygenation. For rectification index and AMPA:NMDA ratio measurements, patch pipettes were filled with an internal solution containing 135 mm CsCl, 2 mm MgCl_6_‐H_2_O, 10 mm 4‐(2‐Hydroxyethyl)‐1‐piperazineethanesulfonic acid (HEPES), 4 mm Mg‐ATP, 0.3 mm Na2‐Guanosine‐5'‐triphosphate (GTP), 10 mm Na2‐phosphocreatine, 1 mm ethylene glycol‐bis(2‐aminoethylether)‐N,N,N',N'‐tetraacetic acid (EGTA), 5 mm QX‐314, and 100 µm spermine. To assess the AMPA:NMDA ratio, the AMPA receptor (AMPAR)‐mediated component was quantified as the peak current amplitude at −70 mV, while the N‐Methyl‐D‐Aspartate Receptor (NMDAR)‐mediated component was defined as the amplitude measured at +40 mV, 50 ms after stimulation, thereby minimizing AMPAR contamination and isolating NMDA receptor‐mediated currents. To analyze the PPF, paired optical stimuli (470 nm, 5 mW, 10 ms duration) were delivered with a 50 ms interstimulus interval. Excitatory postsynaptic currents (EPSCs) were recorded at a holding potential of −70 mV using the same internal solution described above. The paired‐pulse ratio (PPR) was calculated as the amplitude of the second EPSC divided by the first (EPSC_2_/EPSC_1_).

### Electrophysiological Recording for LTP

Artificial cerebrospinal fluid was prepared in advance, with strict control of its osmolarity and pH. Mice were anesthetized with intraperitoneal injections of tribromoethanol and rapidly euthanized to extract the intact brain tissue. The brain was immediately placed in prechilled ACSF, which was continuously bubbled with 95% O_2_ and 5% CO_2_, for stabilization. Vibratome slicing was performed to prepare coronal brain slices with a thickness of 300 µm.

Selected slices were transferred to an incubation chamber, where they were incubated at 37 °C for 30 min, followed by room temperature incubation for 1 h. LTP recordings were conducted using the MED64 (Alpha Med Sciences, Tokyo, Japan) multichannel recording system. High‐frequency stimulation (HFS) was delivered at 100 Hz for four trains, with each train lasting for 500 ms and separated by 10 s intervals.

### Statistical Analysis

The experimental data were presented as mean ± Standard Error of the Mean (S.E.M.) Data analysis and plotting were performed using GraphPad Prism 8.4.2 software (GraphPad Software, San Diego, CA, USA). Potential outliers were identified using the Regression OUTlier (ROUT) method (*Q* = 1%) prior to statistical testing. Normality was tested with the Shapiro–Wilk test, and equal variance was evaluated before analysis of variance analysis. For single comparisons, unpaired or paired two‐tailed *t*‐tests were used, and one‐way or two‐way analysis of variance (ANOVA) followed by Bonferroni posthoc tests were applied for multigroup comparisons. *p* < 0.05 was considered statistically significant. The difference between two groups was assessed using unpaired Student's *t*‐test (two‐tailed), and the variance among multiple groups was assessed by one‐ or two‐way analysis of variance with/without repeated measures followed by the Tukey, Dunnett, Newman‐Keuls, or Bonferroni posthoc test. *p* < 0.05 was considered statistically significant.

## Conflict of Interest

The authors declare no conflict of interest.

## Author Contributions

Y.Z., J.‐X.K., and K.Z. contributed equally to this work. L.‐Q.Z., D.L., and K.S. designed and oversaw the overall study, provided funding, and were responsible for the final review and editing of the manuscript. Y.Z. and J.‐X.K. performed the molecular biological experiments, and bioinformatic analysis. H.‐W.F. performed the electrophysiological recording. Y.Z., J.‐X.K., K.Z., and H.‐W.F. analyzed the data. L.‐L.C., J.‐W.Y., and L.‐J.L., assisted in experimental execution, data acquisition, and initial data analysis. Z.‐G.X., N.B. and F.H. provided critical insights and resources for the study. N.B. assisted and guidded in immunofluorescence staining presented in Figures 1, 2, and 5. All authors reviewed and approved the final manuscript.

## Supporting information



Supporting Information

Supporting Information

## Data Availability

Research data are not shared.
