# Supplementary material for: Epigenetic Regulation of DAPK1 and Netrin‐1 Drives Diabetic Encephalopathy
Source: Adv Sci (Weinh). 2025 Aug 11;12(37):e02535. doi: 10.1002/advs.202502535 (PMC12499468; doi:10.1002/advs.202502535)
Supplement: Supplementary file 1 — Supporting Information [file ADVS-12-e02535-s001.docx]

**
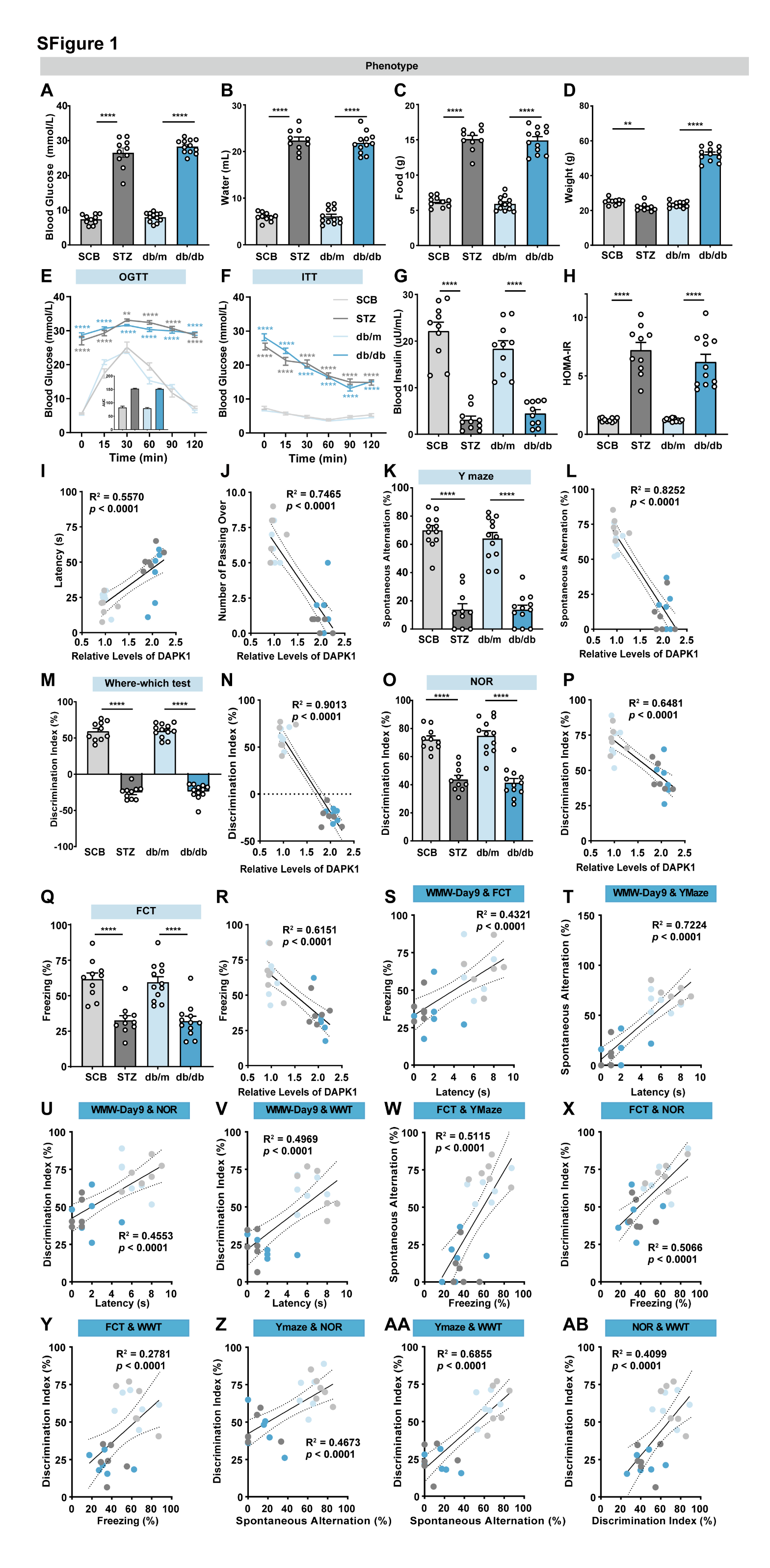
**

**SFig. 1. Cognitive impairments in diabetic mice (Related to Fig. 1).**

(**A** to **D**) Assessment of diabetes-related parameters in diabetic groups (6-month-old SCB-treated mice and db/m mice) and control groups (6-month-old SCB-treated mice and db/m mice): blood glucose levels (**A**), water intake (**B**), food intake (**C**), and body weight (**D**). *n* = 10 to 12 per group.

(**E**) Oral glucose tolerance test (OGTT) results for blood glucose levels at various time points, with the area under the curve (AUC, inset) showing glucose tolerance between diabetic and control groups. *n* = 10 to 12 per group.

(**F**) Insulin tolerance test (ITT) results showing blood glucose levels over time, indicating insulin sensitivity differences across groups.

(**G** to **H**) Blood insulin levels (**G**) and Homeostatic model assessment of insulin resistance (HOMA-IR) (**H**) showing insulin resistance in diabetic mice. *n* = 10 to 12 per group. FGP: Fasting Plasma Glucose; FPI: Fasting Plasma Insulin

(**I** and **J**) Correlation between hippocampal DAPK1 levels and latency on day 7 (**I**) and platform crossings on day 9 (**J**) in the Morris Water Maze. *n* = 24.

(**K** and **L**) The percentage of spontaneous alternations in Y maze (**K**) and its correlation with DAPK1 levels (**L**). *n* = 10 to 12 per group.

(**M** and **N**) Where-which test (WWT) results with discrimination index (**M**) and correlation with DAPK1 levels (**N**). *n* = 10 to 12 per group.

(**O** and **P**) Novel Object Recognition (NOR) test results showing the discrimination index (**O**) and correlation with DAPK1 levels (**P**). *n* = 10 to 12 per group.

(**Q** and **R**) Freezing percentage in the Fearing Condition Test (FCT) (**Q**) and its correlation with DAPK1 levels (**R**). *n* = 10 to 12 per group.

(**S–V**) Correlations between latency in the Morris water maze (WMW, day 9) and freezing percentage in FCT (**S**), spontaneous alternation in the Y maze (**T**), discrimination index in the NOR test (**U**), and discrimination index in the WWT (**V**). *n* = 10 to 12 per group.
(**W–Y**) Correlations between freezing percentage in FCT and spontaneous alternation in the Y maze (W), discrimination index in NOR (X), and discrimination index in WWT (Y). *n* = 10 to 12 per group.
(**Z–AA**) Correlations between spontaneous alternation in the Y maze and discrimination index in NOR (Z), and in WWT (AA). *n* = 10 to 12 per group.
(**AB**) Correlation between discrimination index in NOR and WWT. *n* = 10 to 12 per group.

Data are presented as mean ± S.E.M., and statistical analysis was performed using an unpaired Student’s t-test or one-way or two-way analysis of variance (ANOVA), unless otherwise specified. Linear regression was used for correlation analysis; R² and P values are indicated in each panel. ** *p* < 0.01, *** *p* < 0.001, **** *p* < 0.0001.


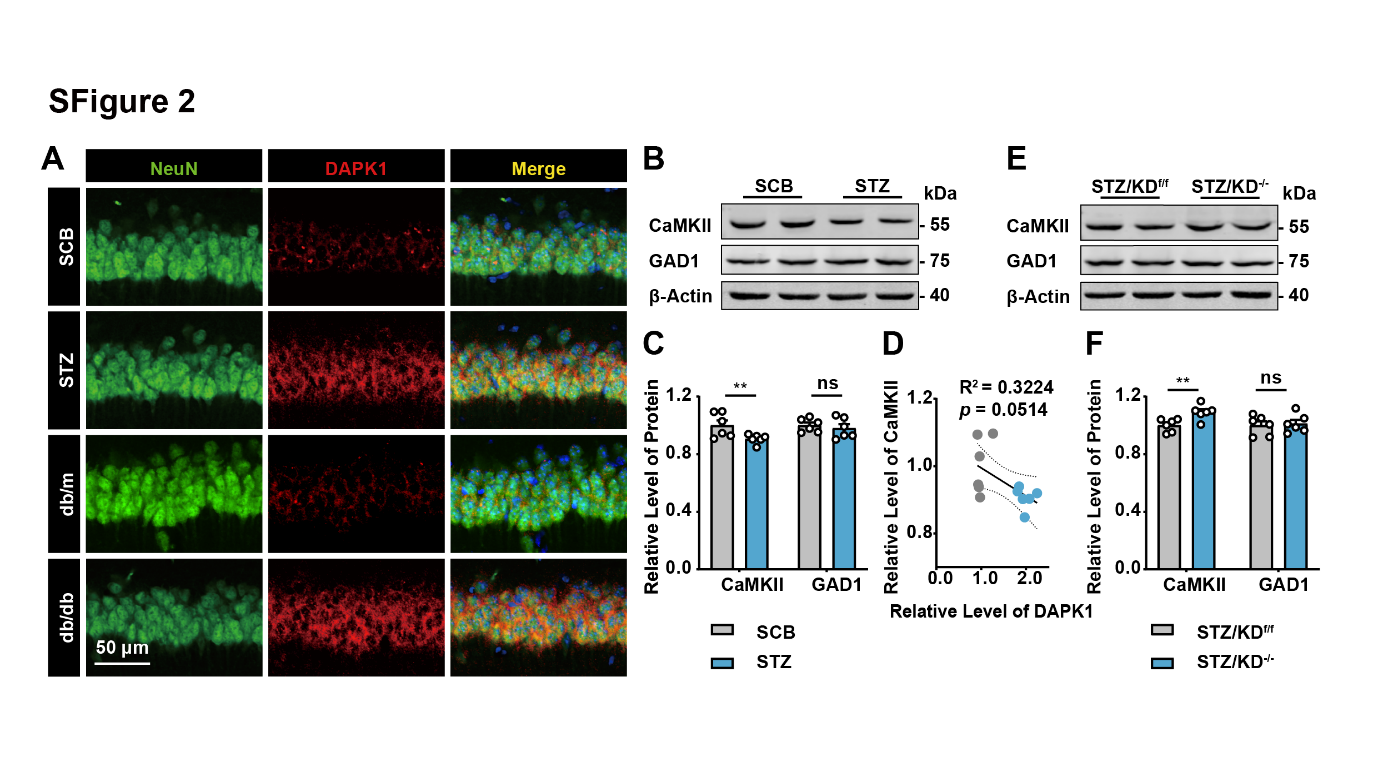


**SFig. 2. DAPK1 deletion partially restores CaMKII and shows inverse correlation trend (Related to Fig. 1).**

(**A**) Representative immunofluorescence images of NeuN (Green) and DAPK1 (Red) immunofluorescent staining in the hippocampus CA1 region of control and diabetic group. Merged images (Yellow) demonstrate the co-localization of NeuN and DAPK1, and nuclei were stained with DAPI (blue).

(**B and C**) Western blot analysis (**B**) and quantification (**C**) of CaMKII and GAD1 expression in hippocampal tissues from control (SCB) and STZ-induced diabetic mice. *n* = 6 per group.

(**D**) Correlation analysis between DAPK1 and CaMKII protein levels across individual samples.

(**E and F**) Western blot analysis (**E**) and quantification (**F**) of CaMKII and GAD1 expression in hippocampal tissues from STZ-induced diabetic mice with DAPK1 conditional knockout (STZ/KD^⁻/⁻^) and littermate controls (STZ/KD^f/f^). *n* = 6 per group.

Data are presented as mean ± S.E.M., and statistical analysis was performed using an unpaired Student’s t-test or one-way or two-way analysis of variance (ANOVA). Linear regression analysis was applied for correlation assessment, with R² and *P* values indicated in the corresponding panel. ** *p* < 0.01, ns: not significant.


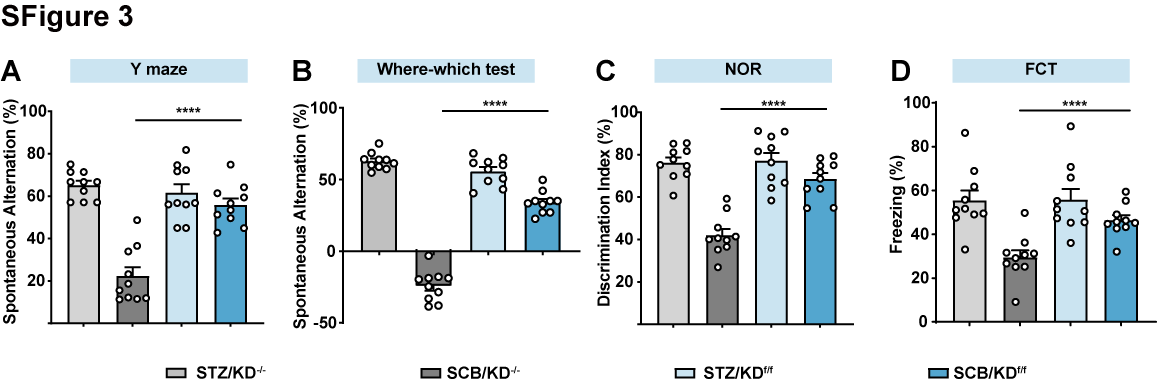


**SFig. 3. Knockdown hippocampus DAPK1 in excitatory neurons rescued cognitive function in diabetes. (Related to Fig. 2).**

(**A**) Y-maze test showing spontaneous alternation percentage, indicating improved spatial working memory in DAPK1^-/-^ mice compared to controls. *n* = 10 per group.
(**B**) Where-which test results displaying spontaneous alternation percentage, further supporting enhanced memory performance in DAPK1^-/-^ mice. *n* = 10 per group.
(**C**) Novel Object Recognition (NOR) test showing the discrimination index, with DAPK1^-/-^ mice demonstrating improved recognition memory. *n* = 10 per group.
(**D**) Freezing behavior (%) in the FCT, with DAPK1^-/-^ mice showing reduced freezing responses in both contextual conditions, indicative of improved cognitive flexibility. *n* = 10 per group.

Data are shown as mean ± S.E.M. Statistical analysis was performed using one-way analysis of variance (ANOVA). **** *p* < 0.0001.


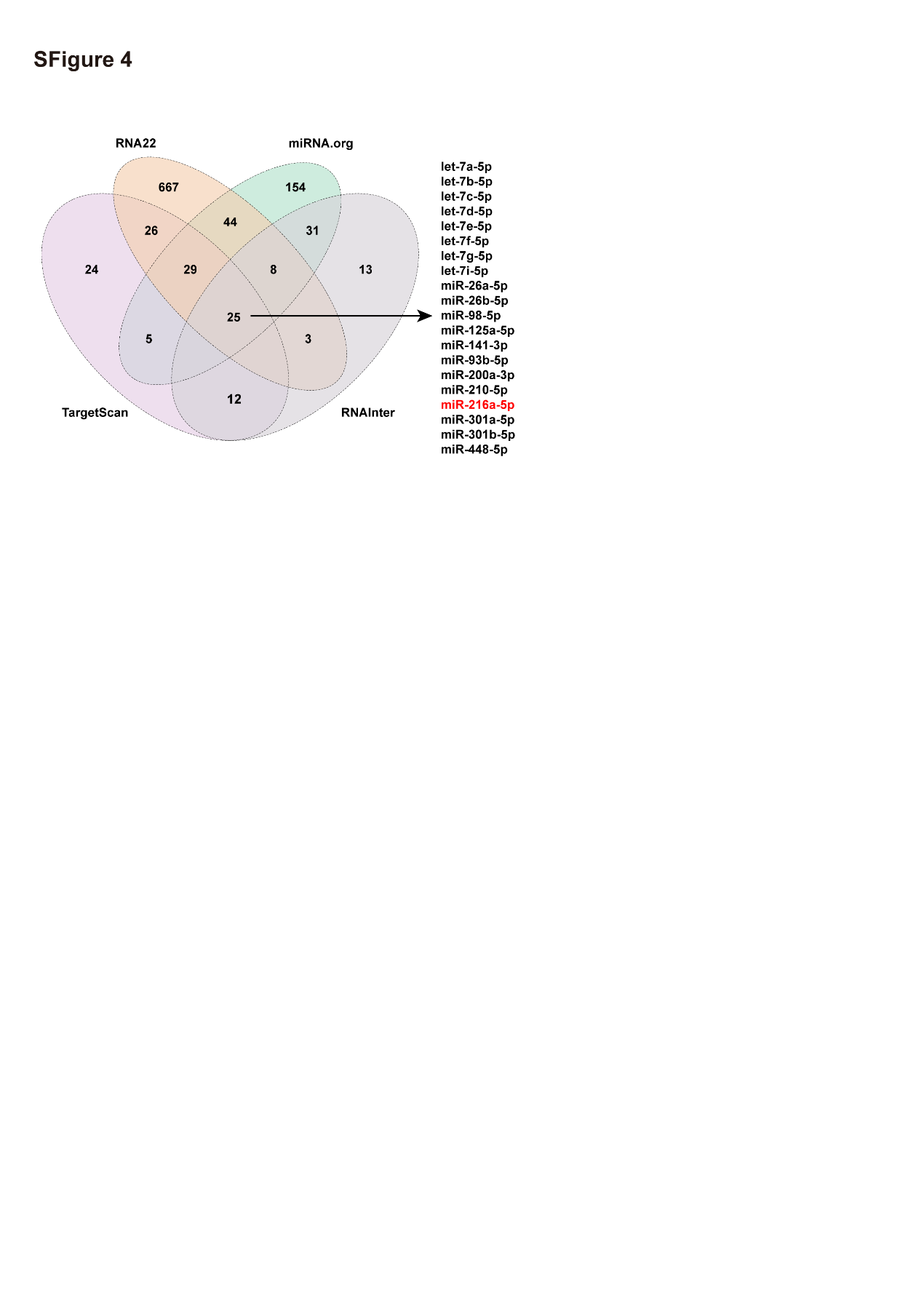


**SFig. 4. Identification of miRNAs targeting DAPK1.**

Venn diagram of predicted miRNAs targeting DAPK1 from four databases: RNA22, miRNA.org, TargetScan, and RNAInter. The overlapping region indicates miRNAs commonly predicted by all four tools, with miRNAs listed on the right representing candidates for further analysis, including miR-216a-5p.


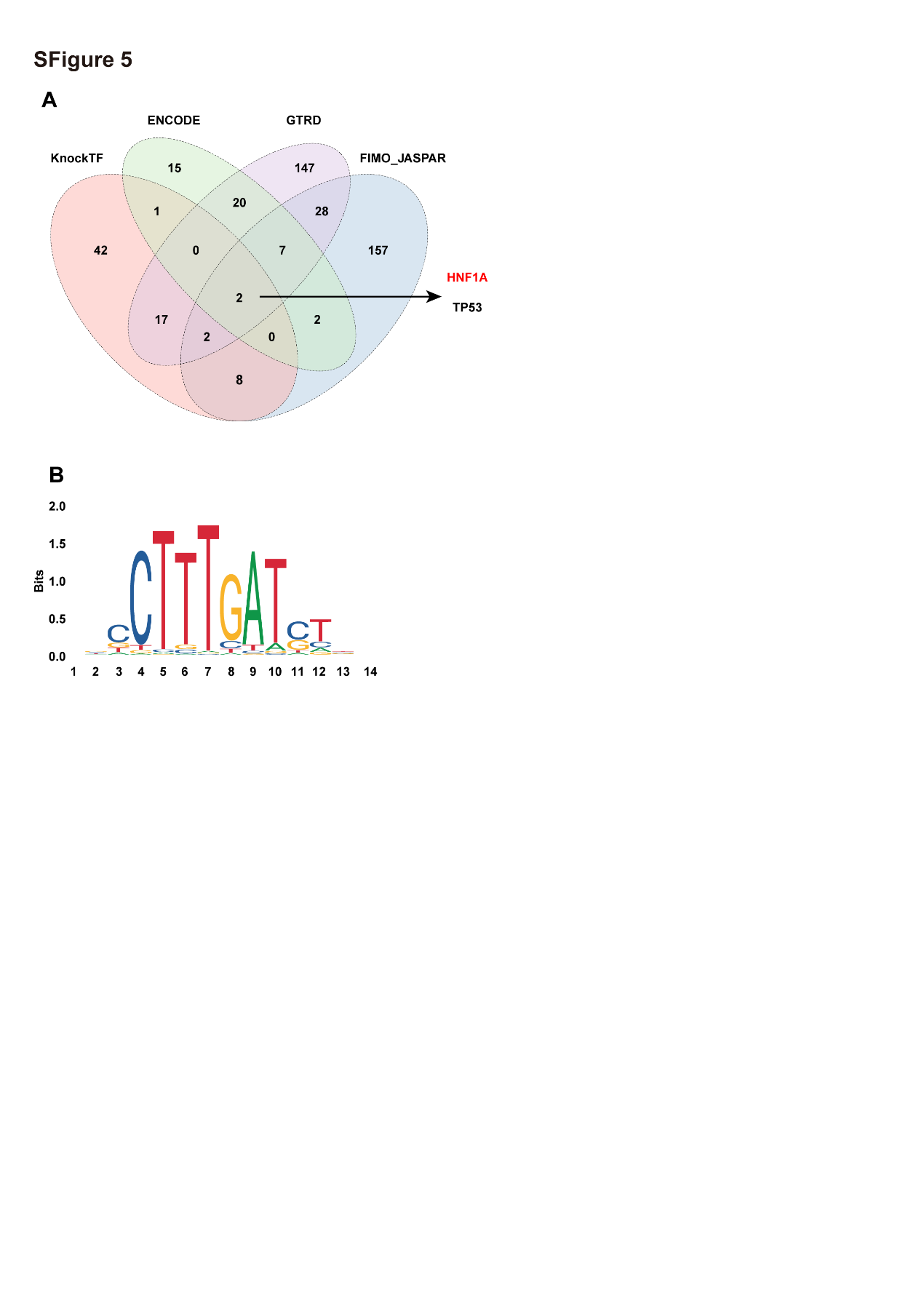


**SFig. 5. Identification of transcription factors and pathways associated with DAPK1 regulation.**
(**A**) Venn diagram showing transcription factors predicted to bind to DAPK1 regulatory regions from four databases: KnockTF, ENCODE, GTRD, and FIMO_JASPAR. The overlapping region highlights HNF1A and TP53 as common transcription factors identified by all four databases.
(**B**) Sequence logo representing the conserved binding motif identified for one of the key transcription factors (HNF1A), displaying the nucleotide frequency at each position.

Data are presented with -log10(P) values to indicate statistical significance of enrichment.


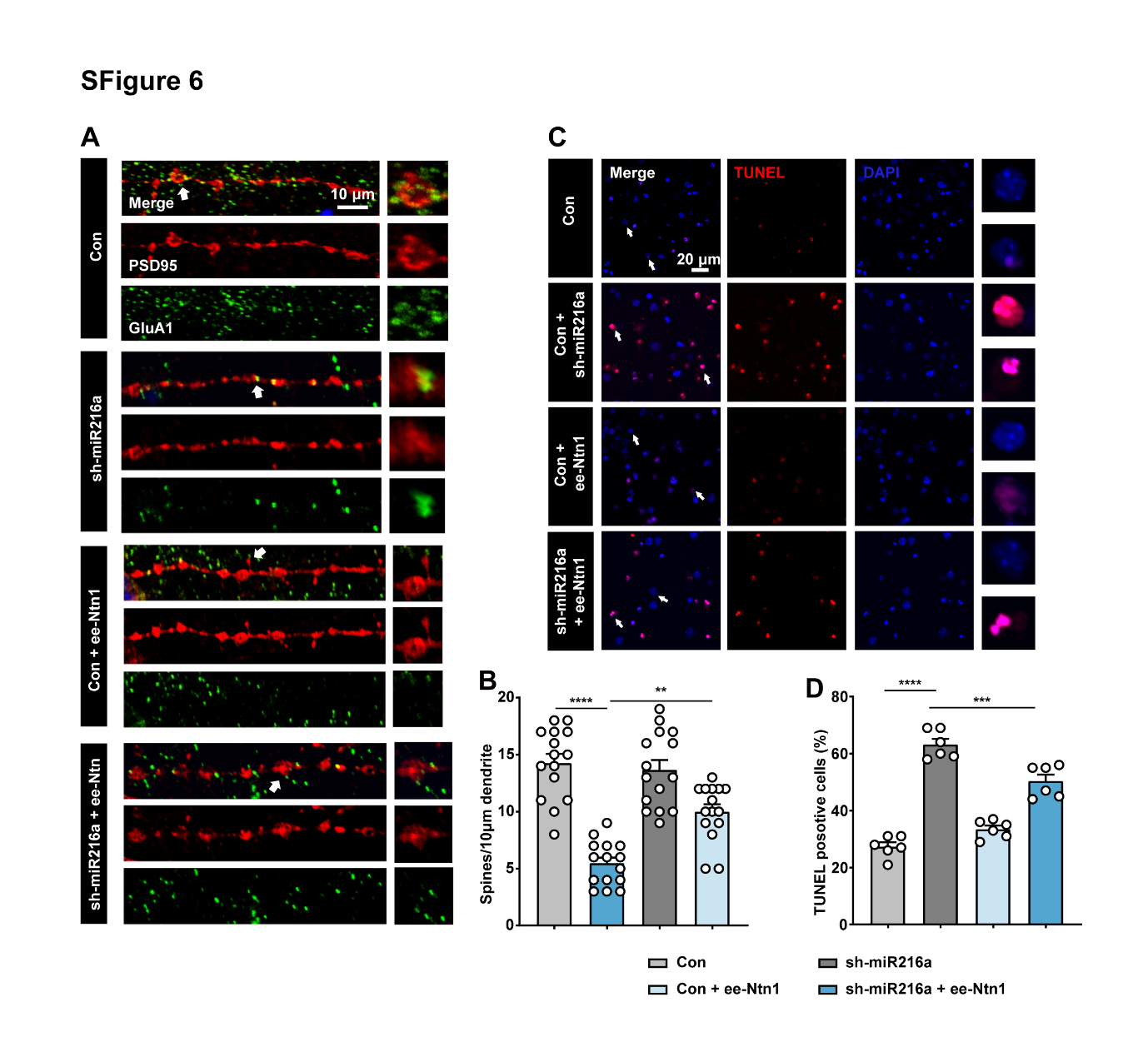


**SFig. 6. Overexpression of Ntn1 rescues synaptic and apoptotic defects induced by miR-216a-5p knockdown.**

1. Representative images of dendritic spines (red: PSD95; green: GluA1) in primary cultured hippocampal neurons under different conditions. Arrows indicate representative co-localized synaptic puncta.
2. Quantification of dendritic spine density (spines per 10 μm dendrite). n = 15 neurons per group.
3. TUNEL staining of primary neurons under the indicated conditions. Arrows point to TUNEL-positive apoptotic cells (red). Nuclei were counterstained with DAPI (blue).
4. Quantification of the percentage of TUNEL-positive cells. n = 6 wells per group.

Data are presented as mean ± SEM. One-way ANOVA followed by Tukey’s post hoc test: ** *p* < 0.01, *** *p* < 0.001, **** *p* < 0.0001.


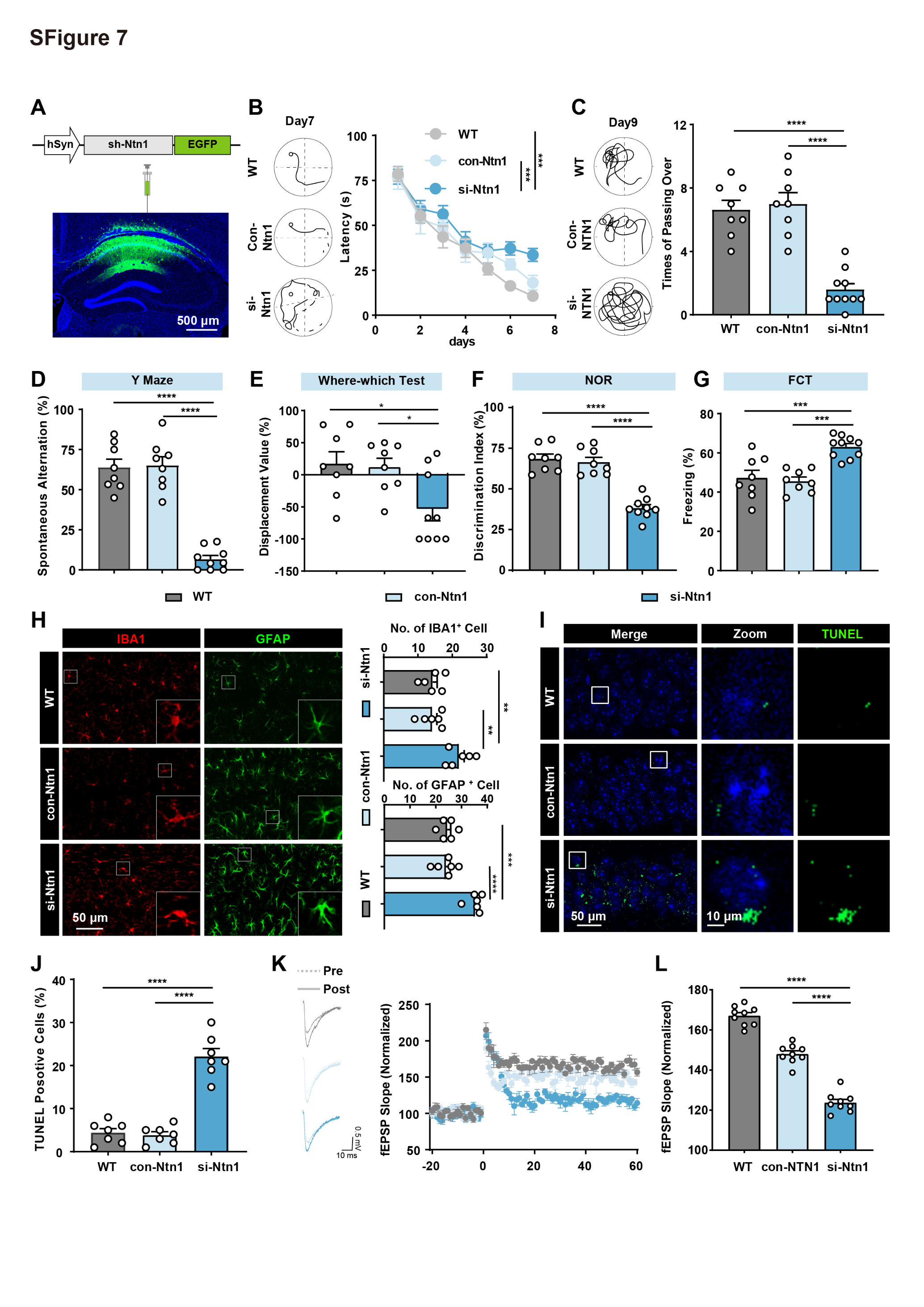


**SFig. 7. Silencing of Netrin-1 induced the diabetic-like memory impairments and diabetic encephalopathy.**

(**A**) Representative confocal image of the neuron-specific Ntn1 shRNA silencing with the virus infection.

(**B**) Representative path traces to the hidden platform on day 7 and latency over 7 days in WT mice group, con-Ntn1 and si-Ntn1 groups. *n* = 8 to 9 per group.

(**C**) Swimming paths on day 9 and number of crossing the platform region on day 9 in the MWM. *n* = 8 to 9 per group.

(**D to G**) Behavioral performance in Y-maze (**D**), WWT (**E**), NOR (**F**), and FCT (**G**) tests following si-Ntn1. *n* = 8 to 9 per group.

(**H**) Representative images and qualification of microglia (IBA1, red) and astrocytic (GFAP, green) activation in the CA1 of the si-Ntn1-treated mice compared to controls. *n* = 6 slices from 3 mice per group.

(**I** and **J**) Representative images (**I**) and quantification (**J**) of apoptotic cells (TUNEL, green) in the hippocampus CA1 of si-Ntn1 and control mice (WT and con-Ntn1). Nuclei were stained with DAPI (blue). The regions marked with white squares in the CA1 area are shown at higher magnification on the right. n = 7 slices from 3 mice per group.

(**K** and **L**) Electrophysiological recordings of fEPSP slope (**K**) and quantitative analysis (**L**) to evaluate the LTP at the CA3-CA1 synapse. n = 9 slices from 3 mice per group

Data are presented as mean ± S.E.M., and statistical analysis was performed using an unpaired Student’s t-test or one-way or two-way analysis of variance (ANOVA), unless otherwise specified. ** *p* < 0.01, *** *p* < 0.001, *****p* < 0.0001.

**Supplementary Tables**

**Supplementary Table 1 List of abbreviations**

| **Abbreviations** | **Full Form** |
| --- | --- |
| 3' UTR | 3' Untranslated Region |
| ACSF | Artificial cerebrospinal fluid |
| AD | Alzheimer’s disease |
| AUC | Area Under the Curve |
| Aβ | Amyloid β-protein |
| BDNF | Brain-derived neurotrophic factor |
| CaMKII | Ca^2+^/calmodulin-dependent protein kinase II |
| CNS | Central nervous system |
| DAPK1 | Death-Associated Protein Kinase 1 |
| DE | Diabetic encephalopathy |
| DG | Dentate gyrus |
| DM | Diabetes mellitus |
| DMEM | Dulbecco's Modified Eagle Medium |
| ee-Ntn1 | Elevated expression Ntn1 |
| FCT | Fear condition test |
| fEPSPs | Field excitatory postsynaptic potentials |
| FISH | Fluorescence in situ hybridization |
| FPG | Fasting Plasma Glucose |
| FPI | Fasting Plasma Insulin |
| G6Pase | Glucose-6-phosphatase |
| GFAP | Glial fibrillary acidic protein |
| GSK-3β | Glycogen Synthase Kinase 3 Beta |
| HEK293 | Human Embryonic Kidney Cells 293 |
| HFS | High-frequency stimulation |
| HNF1A | Hepatocyte nuclear factor 1 homeobox A |
| HOMA-IR | Homeostasis Model Assessment of Insulin Resistance |
| IBA1 | Ionized calcium binding adapter molecule 1 |
| IFG | Impaired fasting glucose |
| ITT | Insulin Tolerance Tests |
| LTP | Long-term potentiation |
| miRNA | Micro RNA |
| MODY3 | Maturity-onset diabetes of the young type 3 |
| mRNA | Messenger RNA |
| MWM | Morris water maze |
| NeuN | Neuron-specific nuclear protein |
| NGF | Nerve growth factor |
| NMDA | N-methyl-D-aspartate |
| NOR | New object recognition |
| Ntn1 | Netrin-1 |
| OGTT | Oral Glucose Tolerance Tests |
| PBS | Phosphate-buffered saline |
| PFA | Paraformaldehyde |
| RIPA | Radioimmunoprecipitation assay |
| RT-qPCR | Quantitative real-time PCR |
| SCB | Saline sodium Citrate Buffer |
| SSC | Saline Sodium Citrate |
| STZ | Streptozotocin |
| TAM | Tamoxifen |
| TP53 | Tumor Protein P53 |
| TUNEL | Terminal deoxynucleotidyl transferase dUTP nick end labeling |
| WWT | Where-which test |

**Supplementary Table 2 Primers used for qPCR**

| qPCR primer | Sequence 5'-3' |
| --- | --- |
| DAPK1-F | CCAGACTGTCTTCCACCAACTC |
| DAPK1-R | TCCTCACACTCACGTTCTCGCA |
| let-7a-5p | TGAGGTAGTAGGTTGTATAGTT |
| let-7b-5p | TGAGGTAGTAGGTTGTGTGGTT |
| let-7c-5p | TGAGGTAGTAGGTTGTATGGTT |
| let-7d-5p | AGAGGTAGTAGGTTGCATAGTT |
| let-7e-5p | TGAGGTAGGAGGTTGTATAGTT |
| let-7f-5p | TGAGGTAGTAGATTGTATAGTT |
| let-7g-5p | TGAGGTAGTAGTTTGTACAGTT |
| let-7i-5p | TGAGGTAGTAGTTTGTGCTGTT |
| miR-124-3p | TAAGGCACGCGGTGAATGCC |
| miR-125a-5p | TCCCTGAGACCCTTTAACCTGTGA |
| miR-141-3p | TAACACTGTCTGGTAAAGATGG |
| miR-193b-3p | AACTGGCCCACAAAGTCCCGCT |
| miR-200a-3p | TAACACTGTCTGGTAACGATGT |
| miR-216a-5p | TAATCTCAGCTGGCAACTGTGA |
| miR-26a-5p | TTCAAGTAATCCAGGATAGGCT |
| miR-26b-5p | TTCAAGTAATTCAGGATAGGT |
| miR-301a-3p | CAGTGCAATAGTATTGTCAAAGC |
| miR-301b-3p | CAGTGCAATGGTATTGTCAAAGC |
| miR-329-3p | AACACACCCAGCTAACCTTTTT |
| miR-342-3p | TCTCACACAGAAATCGCACCCGT |
| miR-362-3p | AACACACCTGTTCAAGGATTCA |
| miR-448-3p | TTGCATATGTAGGATGTCCCAT |
| miR-451a | AAACCGTTACCATTACTGAGTT |
| miR-544-3p | ATTCTGCATTTTTAGCAAGCTC |
| miR-98-5p | TGAGGTAGTAAGTTGTATTGTT |
| U6-F | GATGACACGCAAATTCGTGAA |
| Universal R Primer | GCTGTCAACGATACGCTACG |

**Supplementary Table 3 Antibodies**

| Antibodies | Dilution | Source | | Identifier |
| --- | --- | --- | --- | --- |
| Rabbit anti-β-actin | 1:1000 WB | | Proteintech | Cat# 66009-1-Ig  RRID: AB_2687938 |
| Mouse anti-GFAP | 1:300 IF | | Cell Signaling Technology | Cat# 3670  LOT# 9 |
| Mouse anti-IBa1 | 1:300 IF | | Abcam | Cat# ab283319  LOT# 1007082-1 |
| Mouse anti-NeuN | 1:300 IF | | Cell Signaling Technology | Cat# 94403S |
| CaMKII | 1:200 IF | | Cell Signaling Technology | Cat# 50049  LOT# 1 |
| GAD1 | 1:300 IF | | Sigma-Aldrich | Cat#SAB4501074  UNSPSC Code: 12352203 |
| Rabbit anti-DAPK1 | 1:500 IF  1:1000 WB  1:300 IHC | | Proteintech | Cat# 25136-1-AP |
| Rabbit anti-DAPK1 | 1:500 IF  1:1000 WB | | Cell Signaling Technology | Cat# 3008 |
| Mouse anti-DAPK1 | 1:500 IF  1:1000 WB | | Proteintech | Cat# No.67815-1-lg |
| HNF1A | 1:1000 WB | | Proteintech | Cat # 22426-1-AP |
| p-HNF1A | 1:1000 WB | | Affinity | Cat #: AF7045 |
| Netrin1 | 1:1000 WB  1:500 IF | | Affinity | Cat #: DF8579 |
| IF, Immunofluorescence; WB, Western Blot; IHC, Immunohistochemical staining | | | | |

**Supplementary Table 4 Statistical analysis**

Part 1

| Figure | Statistic method | Comparison | Number (n) | | Statistic results | | Value |
| --- | --- | --- | --- | --- | --- | --- | --- |
| 1B | Two-way ANOVA with Bonferroni's multiple comparisons test | SCB vs STZ | 10, 10 | *p =* 0.0012 Interaction  *p* < 0.0001 Day  *p* < 0.0001 Group | | F (6, 108) = 3.995  F (3.980, 71.63) = 68.63  F (1, 18) = 72.86 | |
|  | Two-way ANOVA with Bonferroni's multiple comparisons test | db/m vs db/db | 12, 12 | *p =* 0.0006 Interaction  *p* < 0.0001 Day  *p* < 0.0001 Group | | F (6, 132) = 4.258  F (4.873, 107.2) = 59.08  F (1, 22) = 85.15 | |
| 1C | unpaired t test | SCB vs STZ | 10, 10 | | *p <* 0.0001 | | t = 9.353, df = 18 |
|  | unpaired *t* test | db/m vs db/db | 12, 12 | | *p <* 0.0001 | | t = 7.978, df = 22 |
| 1E | unpaired t test | SCB vs STZ | 6, 6 | | *p <* 0.0001 | | t = 11.57, df = 10 |
|  | unpaired *t* test | db/m vs db/db | 6, 6 | | *p <* 0.0001 | | t = 23.44, df = 10 |
| 1G  (CA1) | unpaired t test | SCB vs STZ | 6, 6 | | *p <* 0.0001 | | t = 13.76, df = 10 |
|  | unpaired *t* test | db/m vs db/db | 6, 6 | | *p <* 0.0001 | | t = 9.179, df = 10 |
| 1G  (DG) | unpaired t test | SCB vs STZ | 6, 6 | | *p <* 0.0001 | | t = 15.92, df = 10 |
|  | unpaired *t* test | db/m vs db/db | 6, 6 | | *p <* 0.0001 | | t = 8.268, df = 10 |
| 1G  (CA3) | unpaired t test | SCB vs STZ | 6, 6 | | *p <* 0.0001 | | t = 16.39, df = 10 |
|  | unpaired *t* test | db/m vs db/db | 6, 6 | | *p <* 0.0001 | | t = 15.04, df = 10 |
| 1K  (IBA1) | unpaired t test | SCB vs STZ | 6, 6 | | *p <* 0.0001 | | t = 10.03, df = 10 |
|  | unpaired *t* test | db/m vs db/db | 6, 6 | | *p =* 0.0003 | | t = 5.506, df = 10 |
| 1K  (GFAP) | unpaired t test | SCB vs STZ | 6, 6 | | *p <* 0.0001 | | t = 9.402, df = 10 |
|  | unpaired *t* test | db/m vs db/db | 6, 6 | | *p =* 0.0001 | | t = 6.031, df = 10 |
| 1M | unpaired t test | SCB vs STZ | 4, 4 | | *p =* 0.0001 | | t = 8.846, df = 6 |
|  | unpaired *t* test | db/m vs db/db | 4, 4 | | *p =* 0.0003 | | t = 7.475, df = 6 |
| 1O | unpaired t test | SCB vs STZ | 9, 9 | | *p <* 0.0001 | | t = 10.20, df = 16 |
|  | unpaired *t* test | db/m vs db/db | 9, 9 | | *p <* 0.0001 | | t = 14.48, df = 16 |
| S1A | unpaired t test | SCB vs STZ | 10, 10 | | *p <* 0.0001 | | t = 13.65, df = 18 |
|  | unpaired *t* test | db/m vs db/db | 12, 12 | | *p <* 0.0001 | | t = 29.69, df = 22 |
| S1B | unpaired t test | SCB vs STZ | 10, 10 | | *p <* 0.0001 | | t = 20.40, df = 18 |
|  | unpaired *t* test | db/m vs db/db | 12, 12 | | *p <* 0.0001 | | t = 19.51, df = 22 |
| S1C | unpaired t test | SCB vs STZ | 10, 10 | | *p <* 0.0001 | | t = 15.97, df = 18 |
|  | unpaired *t* test | db/m vs db/db | 12, 12 | | *p <* 0.0001 | | t = 15.18, df = 22 |
| S1D | unpaired t test | SCB vs STZ | 10, 10 | | *p =* 0.0018 | | t = 3.659, df = 18 |
|  | unpaired *t* test | db/m vs db/db | 12, 12 | | *p <* 0.0001 | | t = 22.75, df = 22 |
| S1E | Two-way ANOVA with Bonferroni's multiple comparisons test | SCB vs STZ | 10, 10 | *p <* 0.0001 Interaction  *p* < 0.0001 Day  *p* < 0.0001 Group | | F (5, 90) = 41.49  F (2.291, 41.25) = 106.0  F (1, 18) = 222.1 | |
|  | Two-way ANOVA with Bonferroni's multiple comparisons test | db/m vs db/db | 12, 12 | *p <* 0.0001 Interaction  *p* < 0.0001 Day  *p* < 0.0001 Group | | F (5, 110) = 319.4  F (2.094, 46.06) = 558.9  F (1, 22) = 430.1 | |
| S1F | Two-way ANOVA with Bonferroni's multiple comparisons test | SCB vs STZ | 10, 10 | *p <* 0.0001 Interaction  *p* < 0.0001 Day  *p* < 0.0001 Group | | F (5, 90) = 304.1  F (1.933, 34.79) = 649.0  F (1, 18) = 198.4 | |
|  | Two-way ANOVA with Bonferroni's multiple comparisons test | db/m vs db/db | 12, 12 | *p <* 0.0001 Interaction  *p* < 0.0001 Day  *p* < 0.0001 Group | | F (5, 90) = 65.12  F (1.749, 31.49) = 134.7  F (1, 18) = 2233 | |
| S1G | unpaired t test | SCB vs STZ | 10, 10 | | *p <* 0.0001 | | t = 9.380, df = 18 |
|  | unpaired *t* test | db/m vs db/db | 12, 12 | | *p <* 0.0001 | | t = 7.285, df = 18 |
| S1H | unpaired t test | SCB vs STZ | 10, 10 | | *p <* 0.0001 | | t = 9.123, df = 18 |
|  | unpaired *t* test | db/m vs db/db | 12, 12 | | *p <* 0.0001 | | t = 7.780, df = 22 |
| S1I | Linear regression |  | 24 | | *p <* 0.0001 | | R^2^ = 0.5570 |
| S1J | Linear regression |  | 24 | | *p <* 0.0001 | | R^2^ = 0.7465 |
| S1K | unpaired t test | SCB vs STZ | 10, 10 | | *p <* 0.0001 | | t = 10.44, df = 20 |
|  | unpaired *t* test | db/m vs db/db | 12, 12 | | *p <* 0.0001 | | t = 9.537, df = 22 |
| S1L | Linear regression |  | 24 | | *p <* 0.0001 | | R^2^ = 0.8252 |
| S1M | unpaired t test | SCB vs STZ | 10, 10 | | *p <* 0.0001 | | t = 17.07, df = 18 |
|  | unpaired *t* test | db/m vs db/db | 12, 12 | | *p <* 0.0001 | | t = 20.39, df = 22 |
| S1N | Linear regression |  | 24 | | *p <* 0.0001 | | R^2^ = 0.9013 |
| S1O | unpaired t test | SCB vs STZ | 10, 10 | | *p <* 0.0001 | | t = 7.506, df = 18 |
|  | unpaired *t* test | db/m vs db/db | 12, 12 | | *p <* 0.0001 | | t = 7.250, df = 22 |
| S1P | Linear regression |  | 24 | | *p <* 0.0001 | | R^2^ = 0.6481 |
| S1Q | unpaired t test | SCB vs STZ | 10, 10 | | *p <* 0.0001 | | t = 5.475, df = 18 |
|  | unpaired *t* test | db/m vs db/db | 12, 12 | | *p <* 0.0001 | | t = 5.333, df = 22 |
| S1R | Linear regression |  | 24 | | *p <* 0.0001 | | R^2^ = 0.6151 |
| S1S | Linear regression |  | 24 | | *p <* 0.0001 | | R^2^ = 0.4321 |
| S1T | Linear regression |  | 24 | | *p <* 0.0001 | | R^2^ = 0.7224 |
| S1U | Linear regression |  | 24 | | *p <* 0.0001 | | R^2^ = 0.4553 |
| S1V | Linear regression |  | 24 | | *p <* 0.0001 | | R^2^ = 0.4969 |
| S1W | Linear regression |  | 24 | | *p <* 0.0001 | | R^2^ = 0.5115 |
| S1X | Linear regression |  | 24 | | *p <* 0.0001 | | R^2^ = 0.5066 |
| S1Y | Linear regression |  | 24 | | *p <* 0.0001 | | R^2^ = 0.2781 |
| S1Z | Linear regression |  | 24 | | *p <* 0.0001 | | R^2^ = 0.4673 |
| S1AA | Linear regression |  | 24 | | *p <* 0.0001 | | R^2^ = 0.6855 |
| S1AB | Linear regression |  | 24 | | *p <* 0.0001 | | R^2^ = 0.4099 |
| S2C | unpaired t test (CaMKII) | SCB vs STZ | 6, 6 | | *p =* 0.0292 | | t = 2.544, df = 10 |
|  | unpaired t test (GAD1) | SCB vs STZ | 6, 6 | | *p =* 0.5706 | | t = 5.475, df = 18 |
| S2D | Linear regression |  | 12 | | *p* = 0.0514 | | R^2^ = 0.3224 |
| S2F | unpaired t test (CaMKII) | SCB vs STZ | 6, 6 | | *p =* 0.0087 | | t = 3.254, df = 10 |
|  | unpaired t test (GAD1) | SCB vs STZ | 6, 6 | | *p =* 0.7678 | | t = 0.3034, df = 10 |

Part 2

| Figure | Statistic method | Number (n) | | Statistic results | Value |
| --- | --- | --- | --- | --- | --- |
| 2B | Two-way ANOVA with Tukey analysis | 10 per group | *p* = 0.0045 Interaction  *p <* 0.0001 Day  *p <* 0.0001 Group | | F (18, 216) = 2.189  F (4.588, 165.2) = 102.6  F (3, 36) = 43.30 |
| 2C | One way ANOVA, Tukey's post hoc | 10 per group | *p <* 0.0001 | | F (3, 36) = 20.60 |
| 2D  (IBA1) | One way ANOVA, Tukey's post hoc | 6 per group | *p <* 0.0001 | | F (3, 20) = 19.55 |
| 2D  (GFAP) | One way ANOVA, Tukey's post hoc | 6 per group | *p <* 0.0001 | | F (3, 20) = 35.00 |
| 2F | One way ANOVA, Tukey's post hoc | 6 per group | *p <* 0.0001 | | F (3, 20) = 46.61 |
| 2H | One way ANOVA, Tukey's post hoc | 9 per group | *p <* 0.0001 | | F (3, 32) = 75.95 |
| 2I | One way ANOVA, Tukey's post hoc | 10 per group | *p =* 0.6357 | | F (3, 36) = 0.5742 |
| 2J | One way ANOVA, Tukey's post hoc | 10 per group | *p <* 0.0001 | | F (3, 36) = 18.65 |
| 2L | One way ANOVA, Tukey's post hoc | 10 per group | *p <* 0.0001 | | F (3, 568) = 498.5 |
| S3A | One way ANOVA, Tukey's post hoc | 10 per group | *p <* 0.0001 | | F (3, 36) = 33.03 |
| S3B | One way ANOVA, Tukey's post hoc | 10 per group | *p <* 0.0001 | | F (3, 36) = 206.9 |
| S3C | One way ANOVA, Tukey's post hoc | 10 per group | *p <* 0.0001 | | F (3, 36) = 29.41 |
| S3D | One way ANOVA, Tukey's post hoc | 10 per group | *p <* 0.0001 | | F (3, 36) = 10.08 |

Part 3

| Figure | Statistic method | Number (n) | | Statistic results | Value |
| --- | --- | --- | --- | --- | --- |
| 3A | unpaired *t* test | SCB vs STZ  5, 5 | *p =* 0.3586 | | t = 0.9740, df = 8 |
|  | unpaired *t* test | db/m vs db/db  5, 5 | *p =* 0.8799 | | t = 0.1560, df = 8 |
| 3B | unpaired t test for miR-216a-5p | SCB vs STZ  5, 5  db/m vs db/db  5, 5 | *p* < 0.0001  *p =* 0.0075 | | t = 17.08, df = 4  t = 5.004, df = 4 |
| 3D | Two-way ANOVA with Bonferroni analysis | 6, 6, 6, 6 | *p =* 0.0002 Interaction  *p* < 0.0001  *p =* 0.0003 IgG, HNF1A | | F (1, 20) = 21.59  F (1, 20) = 23.52  F (1, 20) = 19.65 |
| 3F | unpaired t test  (for ago-miR-216a) | 4, 4 | *p =* 0.0053 | | t = 4.272, df = 6 |
|  | unpaired t test  (for anta-miR-216a) | 4, 4 | *p =* 0.0012 | | t = 5.574, df = 6 |
| 3H | Linear regression | 30 | *p* < 0.0001 | | R^2^ = 0.7465 |

Part 4

| Figure | Statistic method | Number (n) | Statistic results | Value |
| --- | --- | --- | --- | --- |
| 4A | unpaired t test |  |  |  |
|  | Netrin1 | 3, 3 | *p* = 0.0049 | t = 5.638, df = 8 |
|  | BDNF  NT3  EGF  NT4  FGF  GDNF  CNTF  EPO  IGF1  TNF  PDGF | 3, 3  3, 3  3, 3  3, 3  3, 3  3, 3  3, 3  3, 3  3, 3  3, 3  3, 3 | *p* = 0.0141  *p* = 0.0181  *p* = 0.6449  *p* = 0.2632  *p* = 0.2770  *p* = 0.3250  *p* = 0.5846  *p* = 0.6281  *p* = 0.5652  *p* = 0.9822  *p* = 0.7787 | t = 4.162, df = 8  t = 3.861, df = 8  t = 0.498, df = 8  t = 1.301, df = 8  t = 1.257, df = 8  t = 1.121, df = 8  t = 0.594, df = 8  t = 0.524, df = 8  t = 0.626, df = 8  t = 0.024, df = 8  t = 0.301, df = 8 |
| 4B | unpaired t test | 5, 5 | *p* = 0.0012 | t = 4.879, df = 8 |
| 4C | One way ANOVA, Tukey's post hoc | 5, 5, 5, 5 | *p <* 0.0001 | F (3, 16) = 44.11 |
| 4E | One way ANOVA, Tukey's post hoc | 6, 6, 6, 6 | *p <* 0.0001 | F (3, 20) = 63.64 |
| 4F | Linear regression | 30 | *p =* 0.0002 | R^2^ = 0.4808 |
| 4H | unpaired t test | 5, 5 | *p <* 0.0001 | t = 8.294, df = 8 |
| 4J | unpaired t test | 3, 3  3, 3  3, 3  3, 3 | *p* = 0.0006 DAPK1  *p =* 0.0004 HNF1A  *p =* 0.0001 p-HNF1A  *p =* 0.0001 Ntn1 | t = 10.04, df = 4  t = 10.68, df = 4  t = 8.085, df = 4  t = 5.175, df = 4 |
| 4K | Two-way ANOVA, Tukey's post hoc | 6, 6, 6, 6 | *p <* 0.0001 Interaction  *p* < 0.0001 HNF1A  *p* < 0.0001 MUT | F (2, 12) = 43.41  F (2, 12) = 30.88  F (1, 12) = 352.1 |
| 4L | One way ANOVA, Tukey's post hoc | 3, 3, 3 | *p* = 0.0002 | F (2, 12) = 19.13 |
| S6B | One way ANOVA, Tukey's post hoc | 15, 15, 15, 15 | *p <* 0.0001 | F (3, 56) = 32.41 |
| S6D | One way ANOVA, Tukey's post hoc | 6, 6, 6, 6 | *p <* 0.0001 | F (3, 20) = 80.49 |

Part 5

| Figure | Statistic method | Number (n) | Statistic results | Value |
| --- | --- | --- | --- | --- |
| 5A | One way ANOVA, Tukey's post hoc | 4, 4, 4, 4 | *p <* 0.0001 | F (3, 12) = 52.91 |
| 5B | Two-way ANOVA with Bonferroni analysis | 10, 10, 10, 10 | *p* = 0.6928 Interaction  *p* < 0.0001 Days  *p <* 0.0001 Group | \| F (18,216) = 0.8055 \| \| --- \| \| F (4.036, 145.3) = 53.82 \| \| F (3, 36) = 10.15 \| |
| 5C | One way ANOVA, Tukey's post hoc | 10, 10, 10, 10 | *p <* 0.0001 | F (3, 36) = 28.37 |
| 5D | One way ANOVA, Tukey's post hoc | 10, 10, 10, 10 | *p <* 0.0001 | F (3, 36) = 0.6354 |
| 5E | One way ANOVA, Tukey's post hoc | 10, 10, 10, 10 | *p* = 0.0003 | F (3, 36) = 8.073 |
| 5F | One way ANOVA, Tukey's post hoc | 10, 10, 10, 10 | *p* < 0.0001 | F (3, 36) = 28.08 |
| 5G | One way ANOVA, Tukey's post hoc | 10, 10, 10, 10 | *p* < 0.0001 | \| F (3, 36) = 40.88 \| \| --- \| |
| 5H | (Iba1) One way ANOVA, Tukey's post hoc | 6, 6, 6, 6 | *p* < 0.0001 | \| F (3, 20) = 15.20 \| \| --- \| |
|  | (GFAP) One way ANOVA, Tukey's post hoc | 6, 6, 6, 6 | *p* < 0.0001 | F (3, 20) = 35.02 |
| 5J | One way ANOVA, Tukey's post hoc | 6, 6, 6, 6 | *p* < 0.0001 | \| F (3, 20) = 33.67 \| \| --- \| |
| 5L | One way ANOVA, Tukey's post hoc | 9, 9, 9, 9 | *p* < 0.0001 | F (3, 32) = 123.1 |
| S7B | Two-way ANOVA with Bonferroni analysis | 8, 8, 9 | p = 0.1591 Interaction  p = 0.0385 Group  p < 0.0001 Days | \| F (12, 132) = 1.432 \| \| --- \| \| F (2, 22) = 3.791 \| \| F (3.198, 70.35) = 67.94 \| |
| S7C | One way ANOVA, Tukey's post hoc | 8, 8, 9 | *p <* 0.0001 | F (2, 22) = 28.12 |
| S7D | One way ANOVA, Tukey's post hoc | 8, 8, 9 | *p <* 0.0001 | F (2, 22) = 60.99 |
| S7E | One way ANOVA, Tukey's post hoc | 8, 8, 9 | *p* = 0.0129 | F (2, 22) = 5.332 |
| S7F | One way ANOVA, Tukey's post hoc | 8, 8, 9 | *p <* 0.0001 | F (2, 22) = 43.99 |
| S7G | One way ANOVA, Tukey's post hoc | 8, 8, 9 | *p =* 0.0001 | F (2, 22) = 14.17 |
| S7H | \| (Iba1) One way ANOVA, Tukey's post hoc \| \| --- \| | 6, 6, 6 | *p* = 0.0010 | F (2, 15) = 11.25 |
|  | \| (GFAP) One way ANOVA, Tukey's post hoc \| \| --- \| | 6, 6, 6 | *p* < 0.0001 | \| F (2, 15) = 22.39 \| \| --- \| |
| S7J | One way ANOVA, Tukey's post hoc | 7, 7, 7 | *p <* 0.0001 | F (2, 18) = 66.94 |
| S7L | One way ANOVA, Tukey's post hoc | 9, 9, 9 | *p <* 0.0001 | F (2, 24) = 183.9 |
